# Design of minibinder proteins as universal antagonists against canine and human TNFα

**DOI:** 10.1038/s42003-025-09030-7

**Published:** 2025-11-24

**Authors:** Jun Weng, Zhiyong Wu, Banbin Xing, Yang Hu, Xiaoyu Hu, Meng Mei, Jiaxin Xu, Mengqing Lu, Yibin Chen, Lin Wei, Ke Ming, Zhizheng Wang, Zhuang Li, Zigong Wei

**Affiliations:** 1 State Key Laboratory of Biocatalysis and Enzyme Engineering, School of Life Sciences, Hubei University, Wuhan, Hubei China; 2 National & Local Joint Engineering Research Center of High-throughput Drug Screening Technology, School of Life Sciences, Hubei University, Wuhan, Hubei China; 3 Key Laboratory of Molecular Biophysics of Ministry of Education, College of Life Science and Technology, Huazhong University of Science and Technology, Wuhan, Hubei China; 4 Hubei Jiangxia Laboratory, Wuhan, Hubei China

**Keywords:** Cryoelectron microscopy, Protein design, Tumour-necrosis factors

## Abstract

In this study, we resolve the complex structure of canine TNFα (cTNFα) trimer bound to nanobody molecules at a resolution of 3.1 Å using cryo-electron microscopy. Structural comparison between cTNFα and human TNFα (hTNFα) reveals that the non-conserved residue Phe83 in cTNFα induces a conformational change in a nearby loop, which significantly reduces the binding affinity of cTNFα to nanobody TNF30. By analyzing the simulated complex structures of cTNFα with its receptors and resolved structures of hTNFα in complex with its receptors, we identify a conserved hydrophobic region involved in ligand-receptor interaction on both TNFαs. Five hydrophobic residues within this region are determined as target hotspots to allow de novo computational design of universal minibinders against both canine and human TNFα. Purified top-ranked designs exhibit sub-nanomolar to nanomolar affinity toward cTNFα as well as hTNFα, and show anti-TNFα activities to both TNFαs in cell-based assays that are comparable or superior to the anti-hTNFα activity of TNF30. This work provides a practical approach to generate universal TNFα antagonists as promising lead molecules for the development of potent cross-species biologics applicable in both humans and canines.

## Abstract (web summary)

De novo computational design of minibinder proteins, which achieve sub-nanomolar affinity to both human and canine TNFα by targeting a conserved hydrophobic surface region, enables potent cross-species TNFα antagonism.

## Introduction

Tumor necrosis factor alpha (TNFα) is a pleiotropic cytokine, possessing beneficial functions in immune regulation and host defense, as well as deleterious pro-inflammatory and cytotoxic functions during inflammation^1–3^. Over-expressed TNFα are associated with the development of human immune diseases, including rheumatoid arthritis, psoriatic arthritis, Crohn’s disease, and inflammatory bowel disease^4,5^. Many anti-TNFα drugs have been successfully developed and approved by FDA, most of which are antibodies or antibody-based fusion protein, such as Infliximab^6^, Adalimumab^7^, Golimumab^8^, and Certolizumab pegol^9^. Ozoralizumab is the first approved nanobody drug targeting human TNFα (hTNFα) for the treatment of rheumatoid arthritis because of its excellent efficacy and safety^10^.

Besides in human being, TNFα is widely expressed in various species of animals, and highly conserved among mammal species^11^. Like human beings, dogs develop similar TNFα-involved inflammatory diseases, including osteoarthritis, inflammatory bowel disease, systemic lupus erythematosus, and a variety of autoimmune skin diseases^12–14^, resulting in a great need for canine TNFα (cTNFα) antagonist that can be used in dogs to cure or relief such inflammatory conditions. Considering the high identity between cTNFα and hTNFα, a rational approach is to test FDA-approved hTNFα antibodies on patient dogs. However, only limited and temporary effect of hTNFα antibody Adalimumab has been reported on dogs with lupus erythematosus^15^.

Canonical antibody library screening is an effective method to obtain the high affinity antibody molecules for therapeutic antibody development^16^. In recent years, de novo computational design method is a thriving approach to generate antibody-like minibinder protein with the inherent advantages of excellent thermostability and low immunogenicity^17–19^. A major advantage of computational design is that minibinder proteins could be designed to precisely bind to the particular region of the target protein based on the experimental-determined structure, which provide more possibility and flexibility to obtain specific minibinder against their protein targets, i.e., tunable agonist^20^ and antagonist^17–19^.

TNF30 is an anti-hTNFα nanobody, and key component of the first approved anti-hTNFα nanobody drug Ozoralizumab^21^. In this study, we firstly inspected the affinity of TNF30 to cTNFα. To reveal the details the interaction between nanobody and cTNFα, we determined the complex structure of cTNFα with nanobody by cryo-electron microscopy (cryo-EM). Based on the experimental-resolved and computational complex structures, minibinders targeting on the conserved hydrophobic region in two TNFα were de novo computational designed. Top-ranked designs were expressed and purified. Their affinities and anti-TNFα activities to two TNFα were evaluated by biolayer interferometry and cell-based assays. Our work provided a practical approach to design minibinder proteins as universal antagonist of homologous TNFα proteins in human and canine, which could be promising candidates to develop clinical universal anti-TNFα antagonist for the treatment of human and canine TNFα-related diseases.

## Results

### Optimizing TNF30 for better binding capability with cTNFα to reconstruct cTNFα-nanobody complex structure by cryo-EM

**Fig. 1:**
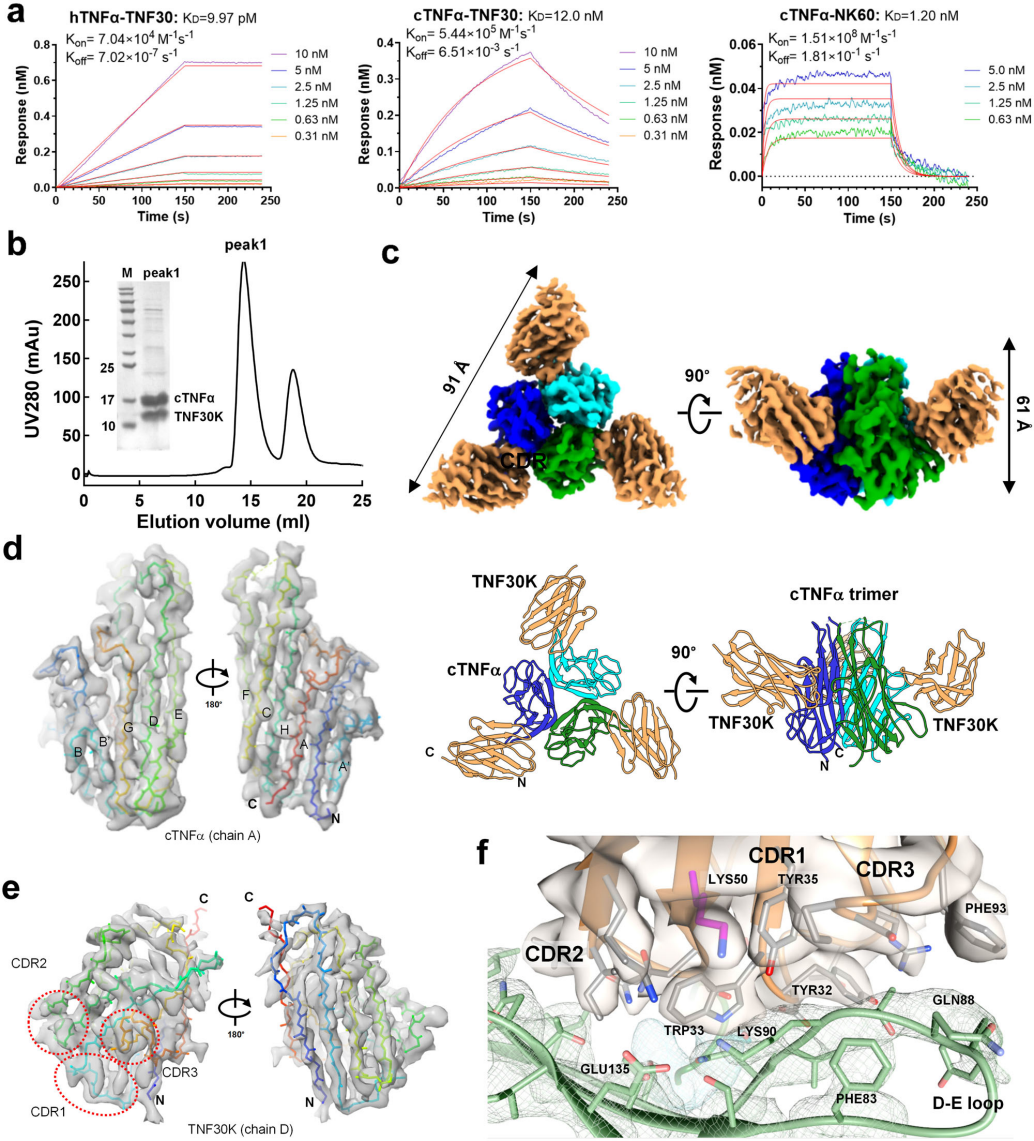
Complex formation of nanobody TNF30 as well as its mutant TNF30K with TNFα and cryo-EM structure of cTNFα-nanobody complex. **a** The binding affinities of nanobody TNF30 and TNF30K to cTNFα or hTNFα were determined using biolayer interferometry (BLI). Reported K_D_s are an average of the K_D_s determined for the two unique interactions. Plots are representative of two independent experiments. **b** Size-exclusion chromatography (SEC) profile of cTNFα-TNF30K mixture on Superdex 200 GL 10/300 column and SDS-PAGE of elution peak corresponding to the complex. ‘M’ represents the protein marker. **c** The overall complex structure of cTNFα-TNF30K. Upper are the top-view and side-view of the 3D reconstruction of cTNFα-TNF30K, with each subunit individually colored. Lower are the cartoon representation of the complex in the same orientation. N and C indicate the N and C termini of the each subunit. **d**,** e** The maps of cTNFα monomer (**d**) and nanobody TNF30K (**e**), and corresponding model fitted in the map. Three complementary determining regions (CDRs) in TNF30K are shown in red dash circles. **f** Local view of the interface between TNF30K and cTNFα. Brown ribbons represent the three CDRs in TNF30K. Residues on the interface are shown with side chains in gray and green color for TNF30K and cTNFα, respectively. Lys50 in TNF30K is shown with side chain in pink color. The cryo-EM density map of TNF30K is shown in surface mode, and map of cTNFα is in mash mode. The contour level is set to 0.30 for density maps presented in (**c**), (**d**), (**e**) and (**f**).

In order to reveal whether anti-hTNFα nanobody TNF30 could bind cTNFα, the recombinant TNF30 protein and cTNFα were expressed in *E. coli* and purified, respectively (Supplementary Fig. 1a, b). Purified TNF30 was mixed with cTNFα in a mole ratio of 2:1 (monomer:monomer), and the mixer was then separated by size-exclusion chromatography (SEC). Two elution peaks were at 14.34 mL and 19.21 mL, and the first elution peak came out obviously earlier than trimeric cTNFα, indicating a mass larger than cTNFα trimer, which was confirmed by SDS-PAGE as the complex of cTNFα-TNF30 (Supplementary Fig. 1c, d). SEC-purified cTNFα-TNF30 complex was applied to prepare the vitrified samples for cryo-EM. After data collection, image processing and map reconstruction, we tried to build up the entire complex structure model based on the reconstructed 3D map but failed. The cryo-EM densities corresponding to TNF30 molecules were rarely poor compared to the densities corresponding to cTNFα trimer, indicating that the binding of TNF30 with cTNFα is not strong enough to form stable complex, leading to the low occupancy of TNF30 in cTNFα-TNF30 complex. BLI was applied to assess the binding affinity of TNF30 to two TNFα proteins, and the affinity of TNF30 to hTNFα is 1000-fold higher than its affinity to cTNFα (Fig. 1a).

To obtain stable nanobody-cTNFα complex for cryo-EM study, screening a mutant of TNF30 to improve the binding affinity to cTNFα should be a practical approach. Based on the modeled structure of cTNFα-TNF30, a detailed energy decomposition analysis on the interface residues based on MM/GBSA method^22^ were processed, and Glu50 was screened out as the candidate residue for mutation (Supplementary Fig. 2). Three representative substitutions, Glu50Lys, Glu50Arg, and Glu50Trp, were constructed and prokaryotically expressed in small-scale for testing, and TNF30-Glu50Lys (TNF30K) showed higher yield and better solubility (Supplementary Fig. 3). The binding affinity of TNF30K to cTNFα is 1.2 nM, which is 10-fold higher than the affinity of TNF30 to cTNFα (Fig. 1a). Afterwards, we scaled up the expression of TNF30K in *E. coli*, and purified TNF30K was applied to prepare complex with cTNFα. SEC profile of the cTNFα-TNF30K mixture showed two elution peaks, and the first peak came out at 14.54 mL, which was consistent with the elution peak of cTNFα-TNF30 complex and confirmed by SDS-PAGE as the complex of cTNFα-TNF30K (Fig. 1b).

The vitrified sample of SEC-purified cTNFα-TNF30K complex was prepared for single particle cryo-EM data collection. After data processing and map reconstitution, we obtained a 3D map with an overall resolution of 3.1 Å with reasonable density for entire hexamer complex, and reconstituted a confident model of cTNFα-TNF30K complex based on the map (Supplementary Fig. 4, Fig. 1c, Table 1). In agreement with the results of SEC profile and SDS-PAGE, three TNF30K bind to a cTNFα trimer to form a heterohexameric cTNFα-TNF30K complex, displaying an equilateral triangular-shaped architecture with a cTNFα homotrimer in the center and three nanobody molecules at the vertex regions of the hexameric complex (Fig. 1c). For each cTNFα monomer, amino acid residues from 9 to 157 was assigned in the model, with two stacks of β-sheets and loop regions between β-strands fitted well into the map except for the E-F loop region (residues 103–112) close to the top of TNFα trimer (Fig. 1d). As to TNF30K, all β-strands and the four loop regions facing inwards to cTNFα trimer fitted into the map well, especially for residues in three complementarity determining regions (CDRs) (Fig. 1e, f). Meanwhile, the cryo-EM densities correspond to the loop regions located at the edge of hexamer are much worse, indicating the corresponding loop regions in TNF30K should be in a dynamic state in solution with flexible conformations (Fig. 1e).

**Table 1:** Cryo-EM data collection, refinement and validation statistics

	cTNFa-TNF30K (PDB: 9JEC, EMD-61412)
**Data collection and processing**	
Magnification	105,000
Voltage (kV)	300
Electron exposure (e^–^/Å^2^)	54
Defocus range (μm)	1.2–2.2
Pixel size (Å)	0.85
Symmetry imposed	C3
Initial particle images (no.)	1,365,774
Final particle images (no.)	543,326
Map resolution (Å) FSC threshold	3.1 0.143
Map resolution range (Å)	3.1–5.0
**Refinement**	
Initial model used (PDB code)	AlphaFold2
Model resolution (Å) FSC threshold	3.1 0.143
Model resolution range (Å)	3.1–5.0
Map sharpening *B* factor (Å^2^)	−193.7
Model composition Non-hydrogen atoms Protein residues Ligands	5985 771 0
*B* factors (Å^2^) Protein Ligand	104.38 0
R.m.s. deviations Bond lengths (Å) Bond angles (°)	0.006 0.655
Validation MolProbity score Clashscore Poor rotamers (%)	1.86 7.48 0.62
Ramachandran plot Favored (%) Allowed (%) Disallowed (%)	92.96 7.04 0

Looking into the interface area between TNF30K and cTNFα, each TNF30K contacts two cTNFα monomers simultaneously, with three CDRs mainly attaching to the concave surface of the outer β-sheet. CDR1 and CDR2 mainly bind to the concave surface of the outer β-sheet covering βD, βE and βG in a TNFα monomer, and two loop regions (A-A’ and E-H loops) in adjacent monomer, while CDR3 attaches to the D-E loop region. The volume data at the interface region showed good enough densities to allow us building a precise structure of cTNFα-TNF30K interface area (Fig. 1f), indicating that the interaction between cTNFα and TNF30K is strong enough to keep the interface in a stable conformation. The single point mutation of Glu50 to Lys in TNF30 was easily confirmed by fitting the side chain of lysine residue to the density map (Fig. 1f).

### Structure comparison of cTNFα-TNF30K with hTNFα-VHH#2

**Fig. 2:**
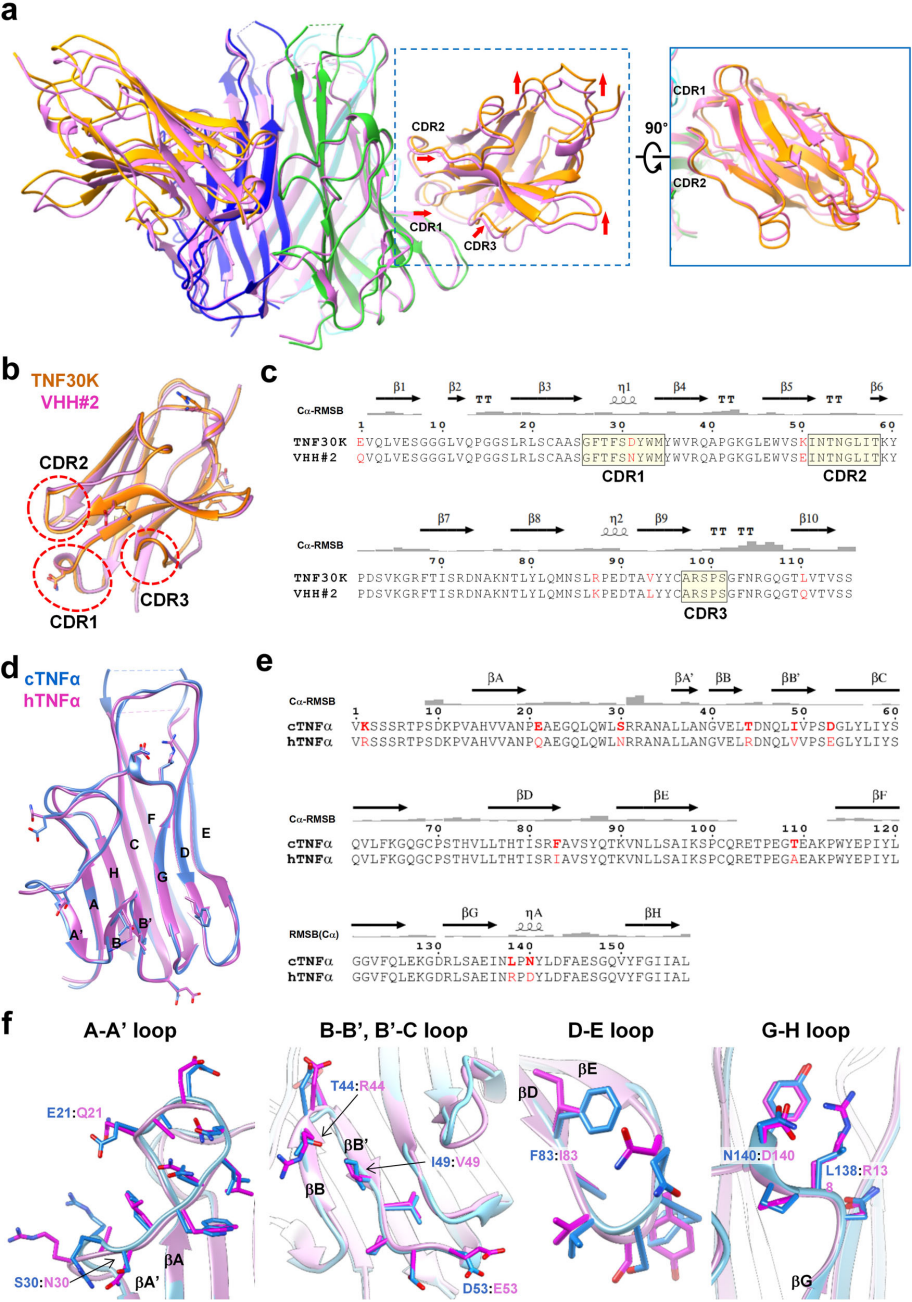
Structural comparison of cTNFα-TNF30K complex and hTNFα-VHH#2. **a** Side view of two complexes superimposed together. For cTNFα-TNF30K, three cTNFα monomers are shown as cartoon in blue, green and cyan, and three TNF30K in brown color. Six subunits in hTNFα-VHH#2 are shown as cartoon in pink. Red arrows indicate the direction of movement of the loop regions in TNF30K compared to corresponding regions in VHH#2. Solid line circled inset presents the top view of overlapped TNF30K and VHH#2 in the complexes. **b**, **c** Comparison of TNF30K and VHH#2 with overlaid structures (**b**) and aligned sequences (**c**). TNF30K is colored in brown, and VHH#2 in pink. The non-conserved residues are shown with side chains in (**b**) or highlighted with red in (**c**). **d**, **e** Comparison of hTNFα and cTNFα monomer with overlaid structures (**d**) and aligned sequences (**e**). cTNFα is colored in blue, and hTNFα in pink. The non-conserved residues are shown with side chains in (**d**) and highlighted with red in (**e**). **f** Structural comparison of loop regions in two TNFα monomers. cTNFα is colored in blue, and hTNFα in pink. Residues within the loop regions are shown with side chains, and non-conserved residues are labeled with corresponding color.

Since no experimental-resolved TNF30 is available, a complex structure of hTNFα-VHH#2 (PDB 5M2J)^23^, in which VHH#2 is an anti-hTNFα nanobody and highly conserved to TNF30 with 4 different residues, was selected for structural comparison with cTNFα-TNF30K. Two hexameric complexes were overlaid together by superimposing cTNFα trimer and hTNFα trimer together (Fig. 2a). Two trimers, cTNFα and hTNFα, show highly identical structures with an average RMSD value of 0.78 Å, indicating no significant overall structural difference between two trimers. As for three TNF30K molecules located at the vertex regions of hexameric complex, their spatial orientations are obviously different with those of VHH#2 in hTNFα-VHH#2 complex, with an average RMSD value of 1.71 Å (Fig. 2a). For each TNF30K, three CDRs are pushed away from the original position of VHH#2, while the loop regions facing outwards of hexameric complex shift a greater distance relative to the position of VHH#2 (Fig. 2a).

To reveal the cause of TNF30K displacement, we firstly compared the structure of TNF30K and VHH#2 (PDB 5M2J.D). Two molecules show highly identical structure with an average RMSD value of 0.858 Å, which is much lower than the value of two proteins in overlaid hexameric complexes, and three CDRs show even higher structural identity (RMSD = 0.55 Å) (Fig. 2b, c), indicating that spatial displacement between TNF30K and VHH#2 in two hexameric complex is more like TNF30K adjusting its orientation as a rigid body to fit in its interaction with cTNFα trimer instead of hTNFα trimer, and the structural difference between cTNFα and hTNFα could be the reason of TNF30K displacement.

To inspect the structural difference between cTNFα and hTNFα, monomers from each complex were extracted and aligned together, with an average RMSD of 0.69 Å. Two stacks of β-sheets are highly conserved and present identical conformation, while loop regions show different conformations with comparatively higher RMSD values (Fig. 2d, e). For 10 non-conserved residues between cTNFα and hTNFα, most of them are located in the loop regions or transition regions between β-strands and loops, i.e., the loops near βA and βA’, the loops near βB and βB’, D-E loop and G-H loop (Fig. 2d, f). These non-conserved residues lead to the diverse conformation as well as various electrostatic and hydrophobic characters in loop regions of two cTNFα monomers.

**Fig. 3:**
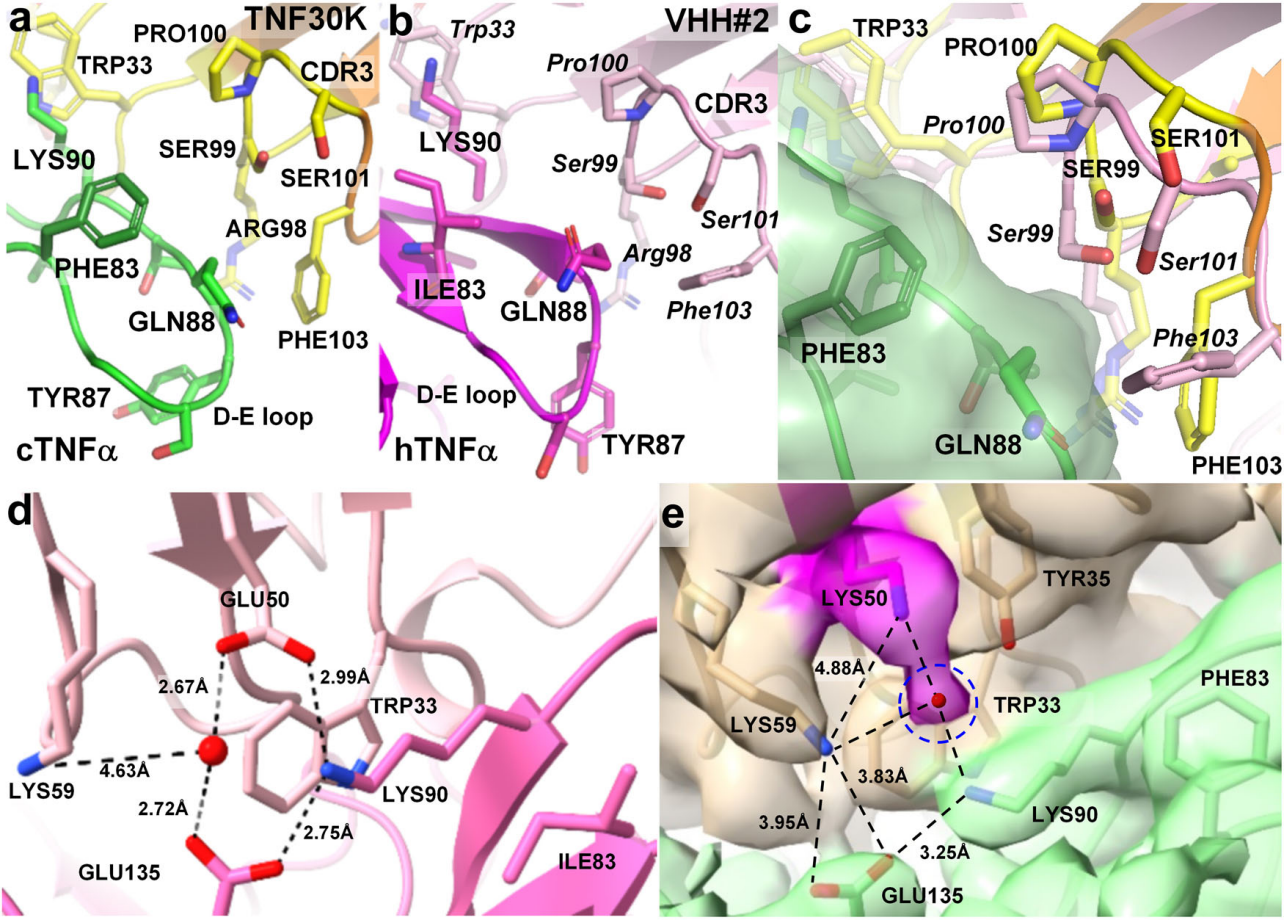
Structural comparison of cTNFα-TNF30K interface and hTNFα-VHH#2 interface. **a**, **b** Interface between D-E loop region and CDR3 in cTNFα-TNF30K (**a**) and hTNFα-VHH#2 (**b**). cTNFα and TNF30K are colored in green and yellow, while hTNFα and VHH#2 in magenta and pink, respectively. **c** An overlay of hTNFα-VHH#2 and cTNFα-TNF30K to illustrate the structure conflict between VHH#2 and cTNFα. cTNFα, TNF30K and VHH#2 are colored in green, yellow/brown and pink, and their residues are labeled with normal (cTNFα, TNF30K) and italicized (VHH#2) fonts, respectively. **d** The salt bridge and hydrogen bond network adjacent to Glu50 on the interface of hTNFα-VHH#2. VHH#2 and hTNFα are colored in pink and magenta, respectively. The water molecule in the water bridge between Glu50 and Glu135 is present as an oxygen atom colored in red. **e** The salt bridge network around Lys50 of cTNFα-TNF30K. TNF30K and cTNFα are colored in tan and green, respectively, and Lys50 in TNF30K is colored in magenta. Cryo-EM map is presented with a contour level of 0.15. The extra density connected with the density of Lys50 was colored with magenta and circled with blue dash line.

As for the interaction between cTNFα and TNF30K, we noticed that the residues within CD3 region (Arg98 to Phe103) of TNF30K are closely attached to D-E loop of cTNFα (Fig. 3a), and non-conserved residue Phe83 is located right at the edge of D-E loop region. Changing from sec-butyl side chain of Ile83 in hTNFα to aromatic group of Phe83 in cTNFα significantly alters the local conformation in D-E loop region. The bulky and hydrophobic aromatic group of Phe83 pushes the Gln88 side chain towards the outside of trimer surface, thereby causing the D-E loop bulge up in cTNFα compared to its original position in hTNFα (Fig. 3b). This conformational change directly leads to a spatial rearrangement of the CDR3 region on the opposite side of the interface (Fig. 3a, b). As shown in Fig. 3c, if TNF30K remains in the position where VHH#2 is located, the side chains of the CDR3 residues, especially for Phe103, would clash with the bulky D-E loop in cTNFα. In resolved complex structure, CDR3 residues in TNF30K recede from the interface compared to the VHH#2 position in hTNFα-VHH#2 complex, leading to the entire TNF30K being slightly farther away from the cTNFα trimer (Fig. 2a).

For the interaction between VHH#2 and hTNFα, CDR1 and CDR2 of VHH#2 bear the major interactions with the concave β-sheet region on hTNFα, including all hydrogen bonds and salt bridges, which is similar to the situation observed in the interaction between cTNFα with TNF30K. Compared to the interactions between hTNFα and VHH#2, two pairs of hydrogen bonds and salt bridge interactions between cTNFα and TNF30K are reconstructed, and the bond lengths of the remaining hydrogen bonds are extended to longer distance between cTNFα and TNF30K (Table 2), suggesting that each bond strength between TNF30K and cTNFα is attenuated, which is consistent with the lower binding affinity of TNF30 to cTNFα compared to hTNFα. In addition, the water bridge around Glu50 in the interface of hTNFα-VHH#2 (Fig. 3d) is disrupted due to the spatial displacement of whole TNF30K from cTNFα and the replacement of Glu50 by Lys in TNF30K. Lys50 has an electrostatic push effect with Lys59, which pushes Lys59 towards cTNFα and strengthens the interaction between Lys59 and Glu135 (Fig. 3e). Meanwhile, a non-proteinous density in the middle of Lys50TNF30K and Lys90cTNFα shows up in the map with a contour level of 0.15, suggesting that an anion (e.g., Cl^-^) might be present at this position and stabilize the strong positively charged microenvironment created by three surrounding lysine residues (Lys50TNF30K, Lys59TNF30K and Lys90cTNFα) (Fig. 3e). Our results indicated that the minor conformational change due to one amino acid difference could significantly affect the interaction between antigen and antibody, and in case of TNF30 and cTNFα, non-conserved Phe83 in cTNFα leads to a 1000-fold decrease of binding affinity of TNF30 to cTNFα compared to hTNFα.

**Table 2:** Interactions between TNFα and nanobody in TNFα-nanobody complexes

cTNFα-TNF30K			hTNFα-VHH#2 (PDB 5M2J)		
cTNFα residues	Dist. (Å)	TNF30K residues	hTNFα residues	Dist. (Å)	VHH#2 residues
**Hydrogen bonds**			**Hydrogen bonds**		
A:THR77 [OG1]	3.86	D:ASN54 [OD1]	A:THR77 [OG1]	2.98	D:ASN54 [OD1]
A:SER81 [OG]	3.42	D:TRP33 [NE1]	A:SER81 [OG]	2.88	D:TRP33 [NE1]
A:LYS90 [O]	3.08	D:TRP33 [N]	A:LYS90 [O]	2.83	D:TRP33 [N]
A:ASN92 [N]	3.57	D:ASP31 [O]	A:ASN92 [N]	2.87	D:ASN31 [O]
B:SER147 [OG]	3.40	D:SER30 [O]	B:SER147 [OG]	2.99	D:SER30 [O]
A:GLU135 [OE2]	3.83	D:LYS59 [NZ]	A:LYS90 [NZ]	2.90	D:GLU50 [OE2]
A:ASN137 [ND2]	3.83	D:LEU56 [O]	A:ASN92 [ND2]	3.27	D:THR53 [OG1]
**Salt bridges**			**Salt bridges**		
A:GLU135 [OE2]	3.83	D:LYS59 [NZ]	A:LYS90 [NZ]	2.99	D:GLU50 [OE2]
A:GLU135 [OE1]	3.95	D:LYS59 [NZ]			

### Designing minibinders towards both cTNFα and hTNFα

Comparative structural analysis of the cTNFα–TNF30K and hTNFα–VHH#2 complexes revealed significant differences between canine and human TNFα within the D-E loop region. This structural divergence explains TNF30’s dramatically lower affinity for cTNFα compared to hTNFα. Additionally, we identified numerous conserved regions in both TNFα isoforms, including the concave β-sheet region on both TNFαs that interacting with nanobody (Table 2). This conservation suggests that a molecule targeting a conserved epitope within the TNFα–TNFR interface, while avoiding interactions with adjacent non-conserved regions, could efficiently block TNFα–TNFR1 interactions for both species. Recent advances in de novo computational minibinder design^17^ provide an ideal approach to develop such minibinder proteins as potential universal TNFα antagonists for canine and human applications. Based on the resolved complex structures of hTNFα with its receptors (PDB:7KPB,3ALQ)^24,25^, it can be found that the elongated ectodomains of receptors bind their corresponding ligand trimers along each of the three clefts formed by neighboring ligand monomers. Considering the high degree of homology and structural similarity between human and canine TNFα proteins, and between the receptor proteins in two species, we hypothesized that cTNFα should interact with its receptors in the same pattern of ligand-receptor interaction in TNF family.

**Fig. 4:**
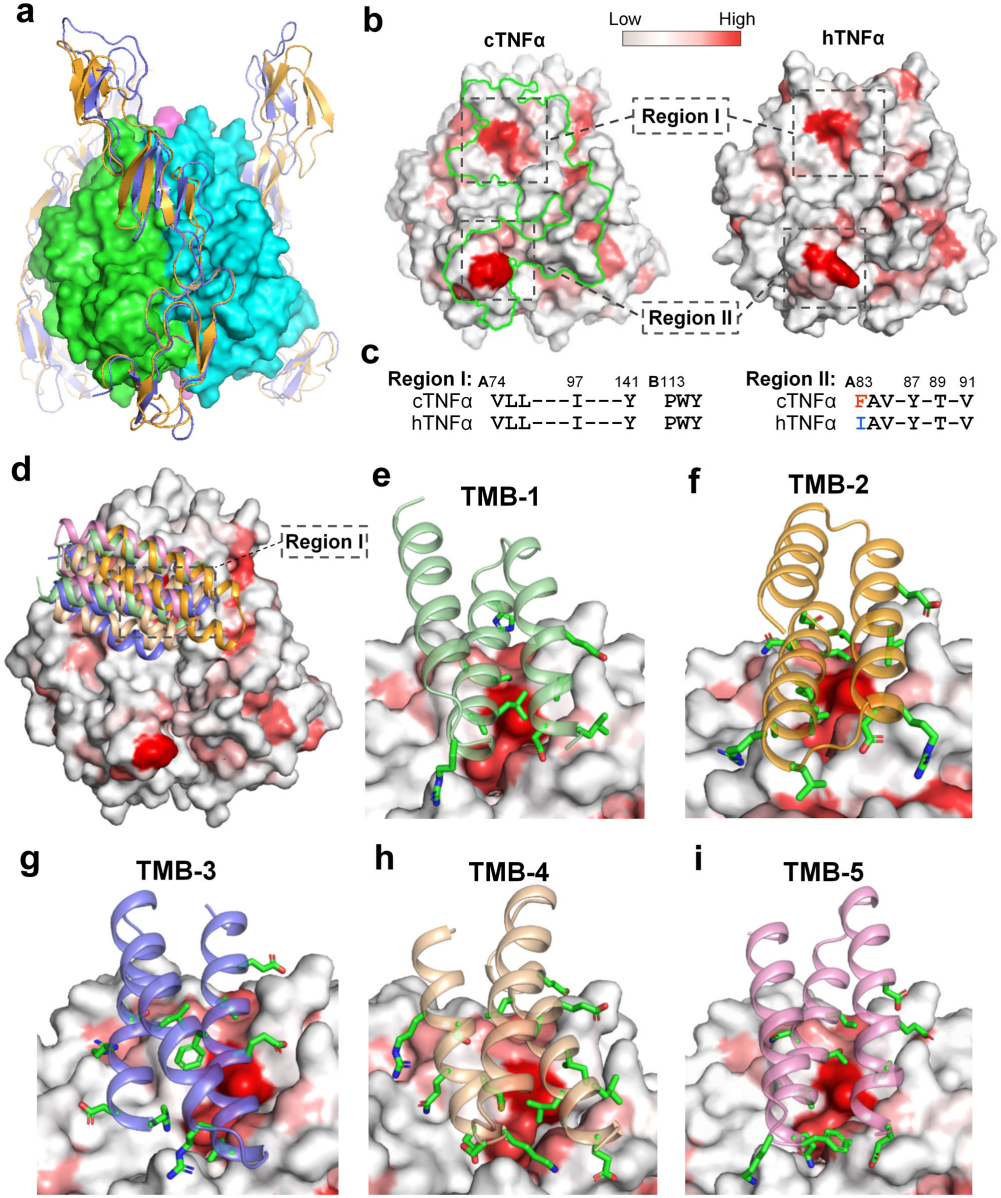
Computational design of minibinders against human and canine TNFα. **a** Overlay of predicted complex structures of cTNFα-cTNFR1 and cTNFα-cTNFR2. Three monomers in cTNFα trimer are colored with green, cyan and magenta in surface mode. cTNFR1 and cTNFR2 are colored in brown and blue in cartoon mode, respectively. **b** Hydrophobic maps of cTNFα (left) and hTNFα (right) in surface mode. Red color represents higher hydrophobicity. Region I and II are two hydrophobic regions on the surface of cTNFα and hTNFα. Green line-circled area represents the overlapping region of two receptor binding sites on cTNFα. **c** Sequence alignment of Region I and Region II in cTNFα and hTNFα. Non-conserved residues are labeled in red and blue color. **d** Overlay of designed minibinders on cTNFα trimer according to the predicted model structures. **e**–**i** Local views of interactions between designed minibinders (TMB-1 to TMB-5) and Region I and adjacent region in cTNFα. Side chains of residues involved in the interaction in minibinder are shown in green color.

In order to identify appropriate regions on cTNFα for computational minibinder design, high-confidence complex models of cTNFα-cTNFR1 (pTM = 0.90/ipTM = 0.89) and cTNFα-cTNFR2 (pTM = 0.89 /ipTM = 0.88) were built using AlphaFold 3^26^ (Supplementary Fig. 5), and then applied to investigate the ligand-receptor interfaces on cTNFα. Looking into the cTNFα trimers in two complex models, the receptor binding interface for cTNFR1 and cTNFR2 are highly consistent, with overlapping region occupying more than 94% and 97% of the interface for each receptor, respectively (Fig. 4a, Supplementary Table 1). Meanwhile, the Rosetta hydrophobic analysis showed that there are two strongly hydrophobic regions (termed “Region I” and “Region II”) on the surface of each monomer in cTNFα trimer, and both are located in the interface area with cTNFR1 and cTNFR2, indicating both regions are involved in the interaction between the ligand and two TNF receptors (Fig. 4b). Region I is located on a concaved cleft region between two monomers close to the top of trimer, and Region II is right on the D-E loop region close to the bottom of trimer. In addition, we found two corresponding hydrophobic regions in hTNFα trimer to regions I and II. These two regions are involved in ligand-receptor interaction with hTNFR1 and hTNFR2 (Supplementary Table 2), but only Region I is conserved and shows a highly consistent conformation in both TNFα proteins, while Region II presents diverse conformation in two TNFα trimers due to a non-conserved residue (Phe83/Ile83) in D-E loop region (Fig. 4c). Because of the hydrophobic feature and comparatively flat surface morphology, Region I was chosen as an ideal region for selecting hotspots for computational design. Five residues in Region I (Val74, Leu75, Ile97 in one monomer and Trp114, Tyr115 in adjacent monomer) were isolated as de novo hotspots for the following computational design. In parallel, 1000 scaffold proteins were placed at the interaction surface to generated 169975 docked configurations. After further optimization and evaluation, five top-ranked minibinders against cTNFα were screened out and listed according to the comprehensive evaluation scores from high to low (Supplementary Table 3). All designs are triple-helix bundles consisting of 56 to 58 residues, with two binding helices interacting hydrophobic region I and its adjacent region, and one additional helix stabilizing two binding helices (Fig. 4d–i). To evaluate the binding capability of five candidate minibinders to hTNFα, the hTNFα-minibinder complex models were generated with MD simulation and optimization (Supplementary Fig. 6), and the binding energy of each minibinder with cTNFα and hTNFα was analyzed according to MM/GBSA and MM/PBGA methods, respectively (Supplementary Table 4). Based on the predicted results, we suggested that the binding affinity of designed minibinders for hTNFα might not be as high as those for cTNFα, nevertheless all minibinders should be able to form stable complex with cTNFα as well as hTNFα.

### Evaluate the binding and activity of minibinders toward both cTNFα and hTNFα

**Fig. 5:**
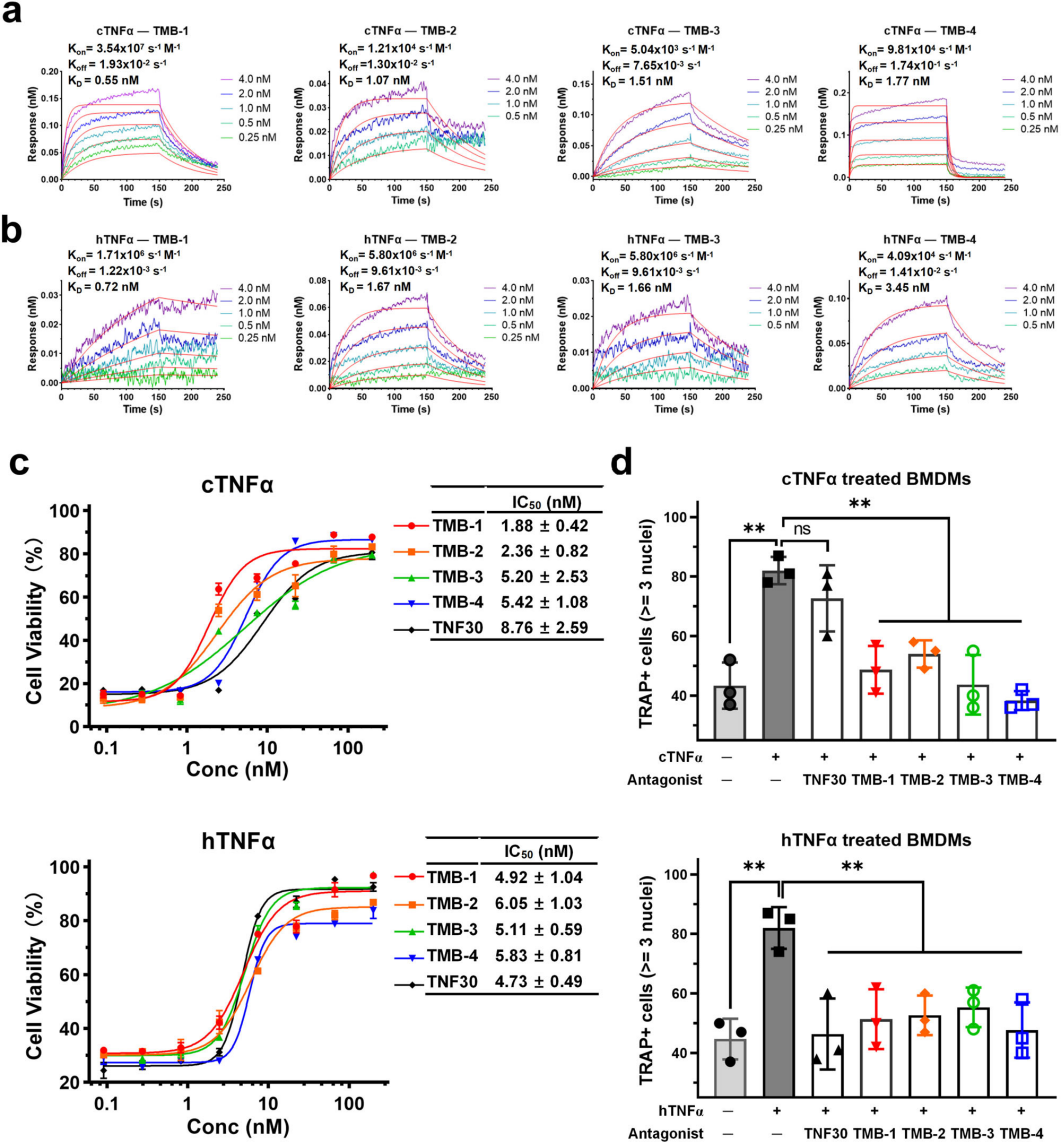
The binding affinity and anti-TNFα bioactivity of minibinders against canine and human TNFα. **a**, **b** The binding affinity of each minibinder to cTNFα (**a**) and hTNFα (**b**) were determined using BLI. Reported K_D_s are an average of the K_D_s determined for the two unique interactions. Plots are representative of two independent experiments. **c** Minibinders block TNFα-mediated cell death of L929 cell line. IC_50_ values reported as mean ± SD of three independent experiments (*n* = 3). Original data are available in supplementary source data file. **d** Minibinders block the promotion effect of TNFα on osteoclastogenesis. TRAP staining-positive cells with at least three nuclei were counted for each treating condition. Data are presented as scatter plots with mean ± SD of three replicates (*n* = 3). Representative TRAP staining images are available in Supplementary Fig. 9. *P* values (**p < 0.01; ns, no significant) define significance.

To assess the binding affinity and anti-TNFα activity of minibinders on both cTNFα and hTNFα, small-scale expression were tested in *E. coli*, and four top-ranked designs (TMB-1 to TMB-4) were successfully expressed and purified while TMB-5 failed in prokaryotic expression (Supplementary Fig. 7). The affinities of purified minibinders to cTNFα was assessed by BLI (Fig. 5a), and TMB-1 had the best binding affinity for cTNFα, which is consistent with the evaluation list (Supplementary Table 3) and binding energy calculation result (Supplementary Table 4). The K_D_ of TMB-1 with cTNFα is 0.55 nM, which is 20-fold higher compared with the affinity of TNF30 to cTNFα (K_D_ = 12.0 nM), but is still much lower than the affinity of TNF30 to hTNFα (K_D_ = 9.97 pM). For each minibinder, the affinity to hTNFα was lower than the value to cTNFα (Fig. 5b), but two values were very close, which is also in agreement with the binding energy calculation result (Supplementary Table 4), indicating that the designed minibinders were able to fulfill our design requirement of binding cTNFα as well as hTNFα, and the comprehensive evaluation to minibinders was reliable.

The mouse fibroblast L929 was a TNFα sensitive cell line with treatment of actinomycin D, and both hTNFα and cTNFα showed dose-dependent cytotoxicity to L929 cells with EC_50_ of 2.22 ng/mL (0.12 nM) and 3.43 ng/mL (0.18 nM), respectively (Supplementary Fig. 8). Therefore, L929 cell assay was applied to assess the anti-TNFα effect of antagonist molecules on both TNFα (Fig.5c). Four minibinders exhibited dose-dependent anti-cTNFα activity with IC_50_ ranging from 1.87 nM to 5.42 nM, which was much better than TNF30’s potency against cTNFα (IC_50_ = 8.76 nM). As for hTNFα, 4 minibinders showed similar potencies with IC_50_ ranging from 4.92 nM to 6.0 nM, comparable to TNF30’s potency against hTNFα (IC_50_ = 4.72 nM). Among 4 minibinders, TMB-1 showed the best potency against both hTNFα and cTNFα, which was close to or even higher than the anti-hTNFα activity of TNF30. As we presented in current study, the binding affinity of TMB-1 for hTNFα and cTNFα are much lower than TNF30’s affinity for hTNFα, and the results of L929 cell assays suggested that we chose the proper and efficiency targeting site on TNFα for computational design, and thus designed minibinder presents TNFα-blocking function with high efficiency.

Furthermore, we detected the anti-TNFα activity in the process of in vitro osteoclastogenesis. It is known that TNFα could synergize with RANKL to promote osteoclastogenesis^27–30^. As shown in Fig. 5d, both hTNFα and cTNFα could promote the osteoclast formation from mouse primary bone marrow-derived macrophages (BMDMs), and minibinder proteins could inhibit the promotion effect of both TNFα on osteoclast formation, whereas TNF30 could only block the promotion effect of hTNFα but have no significant effect on cTNFα. The results of these two cell assays indicate that designed minibinders present excellent neutralizing effect on both TNFα, comparable to or even better than the anti-hTNFα activity of TNF30, which is the core component of clinically proved anti-hTNFα nanodrug Ozoralizumab. In conclusion, by computational design of minibinders targeting the conserved hydrophobic region on both TNFα, our study provided a practical approach to develop universal TNFα antagonist that can efficiently counteract homologous TNFα in human and canine.

## Discussion

In present work, we firstly resolved the complex structure of cTNFα with nanobody by cryo-EM. By comparing the structure of cTNFα and hTNFα, we revealed significant structural differences between human and canine TNFα within the D-E loop region, which dramatically affected the interaction to TNF30. Furthermore, according to the resolved complex structures of hTNFα with monoclonal antibody Adalimumab and Infliximab^31,32^, several loop regions including A-A’ loop, C-D loop and G-H loop are involved in the interaction between hTNFα with antibodies. Various conformations and diverse electrostatic features in these loop regions between two TNFα isoforms would affect their interplaying with monoclonal antibodies. Obviously, canonical antibody screening techniques are able to screen antibodies targeting certain featured sites to fulfill their functions, but cannot methodologically ensure that the screened antibody interacts only with the featured sites but not with adjacent region of featured sites.

In recent years, computational design has emerged as a novel approach to generate small binder proteins from scratch that can interact with certain spots in a target protein, which provided us the opportunity to actively select the binding sites in target proteins and consciously avoid the unwanted regions. Recently, Google DeepMind released an AI system called AlphaProteo which can design novel proteins binding to target molecules^33^. AlphaProteo successfully designed protein binders with sub-nanomolar to nanomolar affinity for diverse targets, including BHRF1, IL-7RA, PD-L1, TrkA, IL-17A, VEGF-A, and so on^33^. However, this system failed to design binders against human TNFα.

In this study, we aimed to design minibinder protein against both cTNFα and hTNFα by precisely targeting minibinder protein to conserved regions in both TNFα^17^. Based on the resolved TNFα complex structures and modeled cTNFα-receptor complex structures, a conserved hydrophobic region (termed “Region I”) was identified on the surface of both TNFα, and then hotspots within this region were determined for minibinder design. BLI results showed that designed minibinders had sub-nanomolar to nanomolar affinities to both cTNFα and hTNFα.

Coincidently, when we investigated the interactions of hTNFα with Adalimumab^31^, Infliximab^32^, and nanobody VHH#2^23^ based on their complex crystal structures, we found that hydrophobic Region I in hTNFα was involved with the interactions between hTNFα and monoclonal antibodies as well as nanobody, indicating that hydrophobic Region I is an ideal binding site for hTNFα antagonist to block the ligand-receptor interaction. In this work, designed minibinders showed excellent anti-TNFα activities to cTNFα as well as hTNFα, indicating that hydrophobic Region I is an ideal targeting site for both human and canine TNFα to develop antagonist.

As highlighted in the Introduction, mammalian TNFα proteins exhibit high sequence and functional conservation. Our comparative analysis of murine and canine TNFα sequences revealed 79% identity and 94% similarity. Critically, our functional data demonstrate cross-species reactivity: both hTNFα and cTNFα show comparable potency in inducing L929 cell cytotoxicity (similar IC_50_ values) and driving osteoclastogenesis in mouse primary BMDMs. These findings confirmed that both hTNFα and cTNFα can bind murine TNF receptors and functionally substitute for murine TNFα. This functional conservation underscores the translational relevance of murine models for evaluating cTNFα antagonists. Therefore, established mouse models employed in human TNFα antagonist development—such as hTNFα transgenic arthritis model, DSS-induced colitis model, and collagen-induced arthritis model—could provide valuable preliminary platforms for assessing minibinder efficacy against cTNFα, particularly when incorporated with species-specific affinity data.

In this study, TNF30 was engaged as an antagonist reference control. TNF30 is the core component of clinically proved anti-hTNFα multivalent nanodrug Ozoralizumab, with excellent anti-hTNFα activity. Among the four designed minibinders, TMB-1 demonstrated nanomolar potency against cTNFα and hTNFα in L929 cell assays, which is comparable to the reported potency of TNF30 against hTNFα (IC_50_ = 0.985 nM)^21^. This performance is notable given that multivalency is a well-established strategy for enhancing nanobody potency. For example, monovalent VHH#2 exhibits an IC₅₀ of 0.748 nM against hTNFα, while its bivalent counterpart achieves significantly greater potency (IC₅₀ = 0.015 nM)²³, even surpassing the sub-nanomolar activities of clinical anti-TNFα antibodies Adalimumab (IC₅₀ = 0.127 nM) and Infliximab (IC₅₀ = 0.144 nM)²³. These results strongly suggest that engineering multivalent minibinders could further enhance their affinity and functional blockade across TNFα isoforms.

Minibinder can be highly expressed in *E. coli*, with advantages of excellent thermostability and low immunogenicity, which can be optimized at the computational design process^17,19^, and hence could be a good choice for a next generation of lower cost protein therapeutics^17^. Minibinders have been designed to target a wide variety of proteins for therapeutic and diagnostic applications, such as receptors involved in signaling and pathogen surface protein, and showed up to sub-nanomolar affinity and in vitro blocking effect to target proteins^17,33^. Furthermore, minibinders targeting integrins αvβ6 and αvβ8 with picomolar affinity can reduce fibrotic burden and improve overall lung mechanics in a lung fibrosis mouse model^18^. A recent research showed that orally administered IL-23R minibinder can reach the therapeutic target past the gut epithelial barrier in the mouse model and shows efficacy better than a clinical anti-IL-23 antibody Guselkumab in mouse colitis^19^. In current study, designed minibinders have low predicted binding affinity to a variety of MHC class II molecules comparing to TNF30, which presented low immunogenicity in mice and human^34,35^ (Supplementary Table 5). Furthermore, Temperature-dependent CD spectra and simulated digestion treatments to TMB-1 showed that this candidate had robust thermostability at high temperature and stable in simulated gastral and intestine fluids within 1 to 2 h (Supplementary Fig. 10). Due to the advantages of small size for diffusion into target tissues with high stability and affinity, such minibinders could be the promising candidate for development as oral biologics against autoinflammatory diseases. Our work suggests that designed minibinders against both human and canine TNFα could be the promising lead molecules for the development of universal antagonist against TNFα-related autoinflammatory diseases in human and canine.

## Methods

### Cell cultures and animals

The murine cell lines L929 was purchased from ServiceBio (Wuhan, China) with authenticated cell line STR analysis report. L929 cells were cultured in complete DMEM medium comprised 10% FBS and 1% penicillin/streptomycin at 37 °C with 5% CO_2_, and the culture medium was refreshed every 2 days. All cells were passaged when they reached ~80–90% confluence.

Primary bone marrow-derived macrophages (BMDMs) were isolated from 3 wild-type C57BL/6 mice (four- to six-week-old, male and female). Mice were anesthetized with isoflurane and humanely euthanized by cervical dislocation. The carcasses were then disinfected with alcohol for 5 min. Following disinfection, the tibia and femur were isolated, and both ends of the epiphyses were cut to the open bone medullary cavity. Bone marrow was flushed with PBS into a 15 mL centrifuge tube using a 1 mL syringe, and collected cell suspension was seeded in 96-well plates. BMDM cells were cultured in complete DMEM and stimulated with M-CSF (50 ng/mL, MCE, USA) for 3 days with 5% CO_2_ at 37 °C.

We have complied with all relevant ethical regulations for animal use. All experimental animal procedures were executed in Hubei University and adhered to institutional guidelines and received approval from the Institutional Animal Care and Use Committee of Hubei University (Hubei, China).

### Expression and purification of TNFα, TNF30 and minibinder proteins in *E. coli*

The codon optimized nucleotides encoding soluble form (Val77 to Leu233) of cTNFα (GenBank: NM_001003244.1) were chemically synthesized, cloned to pET-22a by in-fusion cloning (Vazyme, Nanjing, China), and transformed to *E. coli* BL21 (DE3) cells for culture in LB medium at 37 °C. When OD600 reached 0.6–0.8, cells were induced with 0.5 mM IPTG at 16 °C. After overnight induction, cells were collected and resuspended in ice-cold PBS (10 mM Na_2_HPO_4_, 1.8 mM KH_2_PO_4_, 137 mM NaCl, 2.7 mM KCl, pH 7.4) with 1 mM PMSF, followed by high pressure homogenization. The cell lysate was centrifugated at 10,000 × *g* at 4 °C for 40 min and the supernatant was subjected to Ni-NTA affinity purification. Eluted protein was further purified by gel filtration on a Superdex 200 Increase 10/300 column (Cytiva, Uppsala, Sweden). Residues 77–233 of hTNFα (GenBank: NP_000585.2) were expressed in *E. coli* as a recombinant protein with C-terminal His-tag and purified following the procedures applied for cTNFα.

To express TNF30 and minibinder proteins in *E. coli*, the codon optimized nucleotides encoding TNF30^21^ as well as minibinder proteins (Supplementary Table 3) we designed in this study were chemically synthesized and cloned into pET-22a vector, and then transformed into *E. coli* BL21 competent cells. TNF30 mutants were constructed by site-directed mutagenesis (Vazyme, Nanjing, China). Recombinant proteins were expressed in *E. coli* and then purified following the procedures described ahead. The protein concentration was measured by BCA protein assay kit (Biosharp, Hefei, China). The protein samples were loaded into the wells of 15% SDS-PAGE gels to verify the molecular weight, and evaluate the purity and quantity of the protein.

### Structure modeling and molecular dynamics (MD) calculation

The complex structure model of cTNFα-TNF30 were generated from the complex structure of hTNFα-VHH#2 (PDB 5M2J) by homology modeling, and this initial model was applied for subsequent MD simulations. Three independent simulations were conducted for 100 ns according to the previous procedures^36^ with minor modification (see Supplementary Fig. 2a). The detailed procedures and parameters of MD simulation is provided in supplementary materials. The average coordination of the complex was obtained from the last 5 ns simulation.

The complex models for cTNF-cTNFR1 as well as cTNF-cTNFR2 were generated by AlphaFold 3 web server^26^. The inputs for model construction were the sequences of trimeric cTNFα paired with three copies of the cTNFR1 or cTNFR2 monomer, and the default template library (PDB database up to September 30, 2021) and multiple sequence alignment (MSA) search aligned with the various genomic databases mentioned in the reference paper^26^ were employed. The default automatic random seed was used at the time of job submission. Upon examination of the results, it was determined that the random seeds utilized were 1437417089 and 1516392576 for the hexamer modeling of cTNFα-cTNFR1 and cTNFα-cTNFR2, respectively. The default setting (num_recycles = 10) for the recycle parameter was applied for both complex building. Predicted structures were ranked according to the ranking_score. The highest-ranked model for cTNFα-cTNFR1 had pTM (predicted TM-score for the full structure) = 0.90/ipTM (confidence in the predicted interfaces) = 0.89; for cTNFα-cTNFR2, pTM = 0.89/ipTM = 0.88. These high scores indicate confident predictions (see Supplementary Fig. 4).

### Protein residue optimization based on MD calculation

The binding energies of the cTNFα–TNF30 complex were determined by molecular mechanics/Generalized-Born surface area (MM/GBSA) method using the MMPBSA.py in AMBER22 software. Based on the average coordinate of cTNFα-TNF30 within the last 5 ns period in the 2^nd^ run of MD simulation (Supplementary Fig. 2a), another 5 ns MD simulation was executed, and the last 40 frames (2 ns) were applied for the binding energy calculation using the MM/GBSA technique. The electrostatic desolvation energy was calculated by the Generalized Born (GB) model developed by Tsui and Case^37^. The protein-protein binding interface is relatively hydrophobic, and therefore, the solvent and the solute dielectric constants of GB were set to 80 and 1, respectively^38^. The nonpolar desolation term was estimated from the solvent accessible surface area (SASA): G_np = 0.0072 × SASA + 0.0^39^. Residues participating in the interaction of cTNFα and TNF30 in the model was selected (Supplementary Fig. 2b) and then applied to a detailed energy decomposition analysis by alanine scanning and the binding energy calculation with MM/GBSA method.

To screen out the mutation candidates that could improve the nanobody affinity with cTNFα, similar 5 ns MD simulations and MM/GBSA for energy calculation were carried out for the 17 amino acid substitutions of unfavorable candidate residue on TNF30 for complex formation, and then alanine scanning for the mutated residues were carried out for comparison. and Ser40 and Glu50 were screened out as unfavorable for the interaction (Supplementary Fig. 2c). Furthermore, the RMSD of Glu50 in last 40 frames (2 ns) during MD simulation showed relatively high value, which was consistent to its unfavorable status in the complex structure (Supplementary Fig. 2d). Therefore, Glu50 was selected as the candidate residue for mutation free energy calculation.

### Cryo-EM sample preparation and data acquisition

The cryo-EM grids were prepared by applying 3 μL of protein complex sample to a glow-discharged holey carbon grid (Quantifoil Cu R1.2/1.3, 300 mesh) and blotted for 3 s under 100% humidity at 4 °C before being plunged into liquid ethane using a Mark IV Vitrobot (FEI). Data acquisition was performed on a Titan Krios microscope (FEI) operated at 300 kV with a Gatan K3 detector. EPU software was used for automated data collection according to standard procedures. A calibrated magnification of ×105,000 was used for imaging, yielding a pixel size of 0.85 Å on images. Micrograph were collected with defocus ranging from −1.2 to −2.2 μm and a total dose of 54 e^−^ Å^−2^.

### Cryo-EM image processing and model building

All cryo-EM data processing was performed using program CryoSPARC-v4.0.1^40^. Motion correction was performed using MotionCor2^41^, and CTF was estimated with CTFFIND4^42^. Complex particles were automatically picked and extracted from the full dataset of micrographs. The extracted particles were processed for three rounds of 3D classifications using the initial model generated by CryoSPARC as the reference. One of the 3D classes showed good secondary structural features and their particles were selected and re-extracted. After 3D refinement with *C*_3_ symmetry and particle polishing using CryoSPARC, the final 3D reconstruction of the complex yielded an EM map with a resolution of 3.1 Å.

De novo atomic model building was performed in Coot^43^ based on the 3.1 Å resolution density map of cTNFα-TNF30K complex. The complex structure of cTNFα-TNF30 predicted by homology modeling was applied as the initial model, and was iteratively adjusted and refined against summed maps using Real-space refinement in PHENIX package^44^ and Coot, with secondary structure restraints and non-crystallography symmetry applied. All of the figures were prepared in PyMoL (http://www.pymol.org) or UCSF ChimeraX^45^.

### De novo minibinder protein designing and optimization

The structure of trimeric cTNFα resolved in this study was applied for minibinder designing. The protein binders was computational designed according to the approaches based on the rotamer interaction field (RIF) and Rosetta-based universal binder design procedures as previously described^17,46^ with several modification. Prior to binding protein design, we first analyzed the surface hydrophobicity of the cTNFα and hTNFα trimeric proteins using SAP_constraint@Rosetta, in which SAP score indicates the hydrophobic feature of each residue in proteins that determines their tendency to aggregate^17,47^. Conserved hydrophobic residues involved in ligand-receptor interaction (VAL74, LEU75, ILE97, TRP114, TYR115) were selected as de novo hotspots to generate the RIF residues (disembodied amino acid side chains), side chain atoms of which form favorable interactions with the given cTNFα residues^17^. In parallel, 1000 scaffold proteins (inert de novo-designed proteins with experimentally validated stability) were roughly placed at the desired cTNFα trimers interaction surface using PatchDock. After RIF generation and initial scaffold placement, scaffolds were docked with higher resolution at the interaction surface such that the backbone atoms of the hotspots were matched with appropriate backbone atoms of each scaffold protein, replacing the amino acid previously at that scaffold position. All other scaffold residues, previously computationally optimized for the lowest monomer free energy, were retained. This step generated 169,975 docked configurations.

Each docked configuration was input into a Rosetta design protocol to optimize additional scaffold residues at the target interface for high-affinity binding. Only scaffold side chains within 10 Å of the target surface were allowed to mutate. Scaffold sidechains at surface positions further than 10 Å were not allowed to mutate, but were allowed to optimize rotamer conformation. Target residues within 10 Å of the scaffold were allowed to optimize rotamer conformation. All target and scaffold backbone atoms, all scaffold monomer core side chains, and target side chains further than 10 Å from the scaffold were not allowed to move.

Designed target-minibinder complexes were filtered on metrics thought to predict high-affinity binding, including binding energy, shape complementary of the minibinder to the target surface, score per residues, total SAP score and inhibitor SAP score. After screening, the top five binding proteins were further evaluated. Further virtual docking screen yielded the top-ranked binder against cTNFα trimer, and finally screened out the top 5 mini-binders. To evaluate the binding capability of five candidate minibinders to hTNFα as well as cTNFα, MD simulations of the complexes of minibinders with hTNFα or cTNFα were performed by using the AMBER force field of GROMACS 2019.6 software. Three hundred snapshot structures for each complex were extracted from the smooth MD trajectory at equal intervals for binding free energy calculation.

### Biolayer interferometry

Quantitative assessment of binding affinity was performed using biolayer interferometry on an Octet RED96 (Sartorius). Biotinylated target protein (30 nM cTNFα or hTNFα) was immobilized on streptavidin-coated sensor tips in binding buffer (10 mM HEPES pH 7.4, 150 mM NaCl, 3 mM EDTA, 0.05% Tween20 and 1% BSA) for 15 min. Analyte proteins (minibinders and nanobodies) were diluted from concentrated stocks into the binding buffer. BLI sensor tips were sequentially dipped into buffer only wells (baseline), and then into minibinder/nanobody wells (association), and back into blank buffer wells (dissociation). Data were analyzed and processed using Octet Analysis Studio v.13.0.1.35.

### Neutralizing potency measured in L929 cell-based assay

The TNFα sensitive mouse fibroblast cell line L929s was utilized for measuring the anti-TNFα activity of the selected nanobodies and minibinders. L929 cells were grown until nearly confluent, plated out in 96-well microtiter plates at 5000 cells per well, and incubated overnight. Actinomycin D was added to the cells at a final concentration of 1 µg/mL. Serial dilutions of the nanobodies to be tested were mixed with a cytotoxic concentration of TNFα (10 pM). After incubation for 30 min at 37 °C, this mixture was added to the plated cells and incubated for 24 h at 37 °C. Cell viability was determined by using CCK8 kit. Dose–response curves and EC_50_ values were calculated with GraphPad Prism.

### BMDM differentiation and TRAP staining

Isolated BMDM cells were cultured in complete DMEM and stimulated with M-CSF (50 ng/mL, MCE, USA) for 3 days with 5% CO_2_ at 37 °C. After 3 days, change the medium to complete DMEM with 50 ng/mL M-CSF, 50 ng/mL RANKL, 10 ng/mL TNFα and 100 ng/mL of minibinders or nanobody TNF30. Change the medium every 2 days. BMDM derived osteoclast cells should be ready in 5 to 6 days.

To evaluate osteoclast differentiation, cells were stained for tartrate-resistant acid phosphatase (TRAP) following published protocols^48^ with minor modification. Fixed cells were incubated with TRAP stain solution at 37 °C for 1 h, and then counterstained with Fast Green. Osteoclast cell number was counted under a light microscope at 200⨯ magnification (Supplementary Fig. 9). TRAP-positive multinucleated cells with at least 3 nuclei were scored as osteoclasts.

### Statistics and reproducibility

Statistical analyses were carried out using a GraphPad Prism (version 9.0) software. The two-tailed Student’s t-test was used for comparison between treatment and control groups in cell-based assays, and *p* < 0.05 was considered significant. *p < 0.05; **p < 0.01; ns, not significant (*p* > 0.05). All values were reported as mean ± SD of three replicates (*n* = 3) unless otherwise indicated.

## Supplementary information

Supplementary Information

Description of Additional Supplementary Files

Supplementary Data 1

reporting-summary

## Data Availability

The structure of cTNFα-nanobody complex has been deposited in the Electron Microscopy Data Bank (EMDB) with the accession number EMD-61412, and Protein Data Bank with the accession number PDB ID 9JEC. All experimental data, including source data of figures are provided in the manuscript and supplementary data set file.
