# Supplementary material for: Design of minibinder proteins as universal antagonists against canine and human TNFα
Source: Commun Biol. 2025 Nov 24;8:1639. doi: 10.1038/s42003-025-09030-7 (PMC12644665; doi:10.1038/s42003-025-09030-7)
Supplement: Supplementary file 1 — Supplementary Information [file 42003_2025_9030_MOESM1_ESM.pdf]

Supplementary materials for

Design of minibinder proteins as universal antagonists against canine and human TNFα

Jun Weng, Zhiyong Wu, Banbin Xing, Yang Hu, Xiaoyu Hu, Meng Mei, Jiaxin Xu, Mengqing Lu, Yibin Chen, Lin Wei, Ke Ming, Zhizheng Wang, Zhuang Li, and Zigong Wei

This file includes:

Supplementary materials for molecular dynamics (MD) details of building complex model

Supplementary Figures 1 to 10

Supplementary Tables 1 to 6

Supplementary materials for molecular dynamics (MD) details of building complex model

All MD simulations were conducted using the GROMACS 2019.6 software package[1], employing the AMBER14SB_parmbsc1 force field in combination with the TIP3P water model[2,3]. The system was neutralized, and the simulation was performed at pH 7.0. Detailed parameters for each simulation system are provided in Supplementary Table 6.

Each system was equilibrated in two successive stages. The first stage consisted of a 100 ps simulation under constant volume and temperature (NVT ensemble) at 300 K, with positional restraints applied to the protein. This was followed by a 100 ps simulation under constant pressure and temperature (NPT ensemble) at 1 atm and 300 K, during which the restraints were maintained[4,5]. Throughout both equilibration stages and the subsequent 100 ns production run, all bond constraints—including those involving hydrogen atoms—were enforced using the LINCS algorithm[6].

Long-range electrostatic interactions were treated using the particle mesh Ewald (PME) method, with a real-space cutoff of 1.2 nm for nonbonded interactions[7,8]. Temperature and pressure were maintained using the velocity-rescale temperature coupling scheme and the Parrinello–Rahman pressure coupling algorithm, respectively. The integration time step was set to 2 fs, and simulation data were recorded every 10 ps for subsequent analyses.

Each production MD simulation was performed for 100 ns under constant temperature (300 K) and constant pressure (1 atm). For statistical robustness, each protein complex system was independently simulated in triplicate using different initial velocities and random seeds.

Trajectory analyses were performed using GROMACS built-in tools and custom Python scripts.

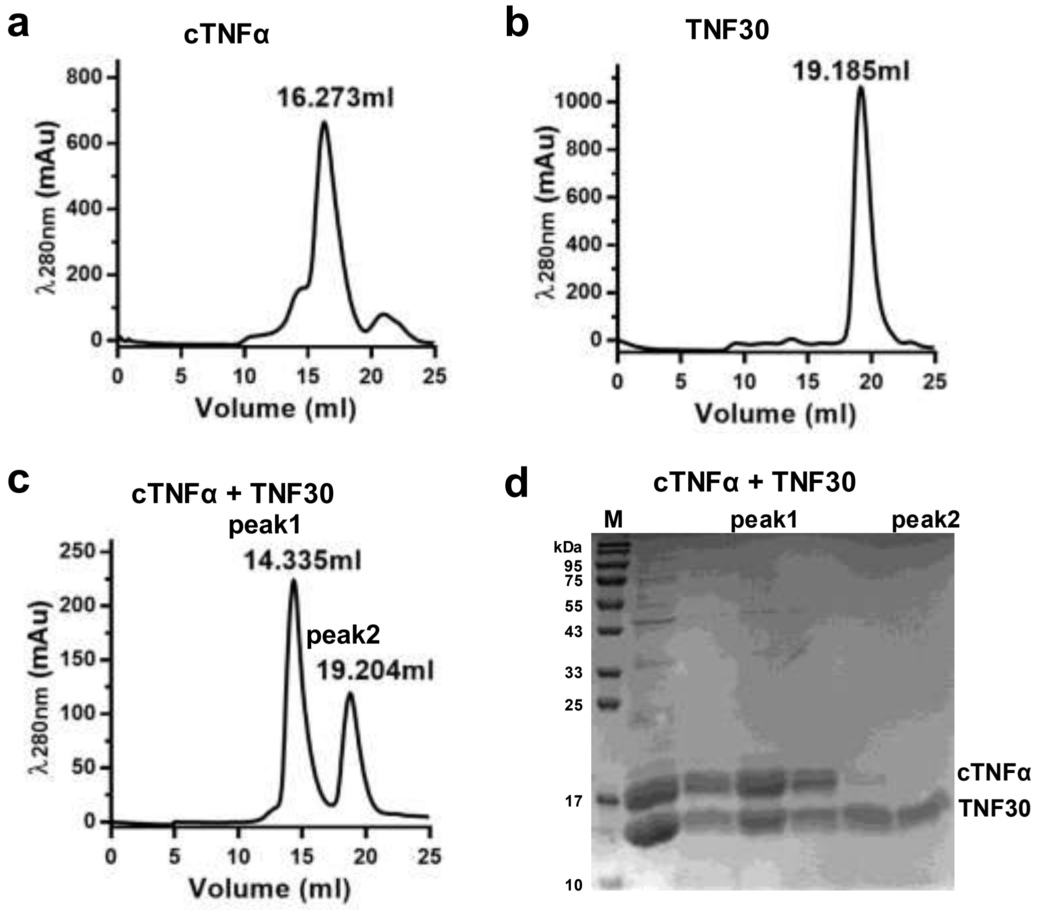

Supplementary Fig. 1: Complex formation of canine TNFα (cTNFα) with nanobody TNF30. **a-c** Size-exclusion chromatography (SEC) profiles of cTNFα (**a**), TNF30 (**b**) and mixture of TNF30 and cTNFα (**c**) on Superdex 200 GL 10/300 column. **d** SDS-PAGE profiles of corresponding SEC peaks of cTNFα-TNF30 complex. 'M' represents the protein marker.

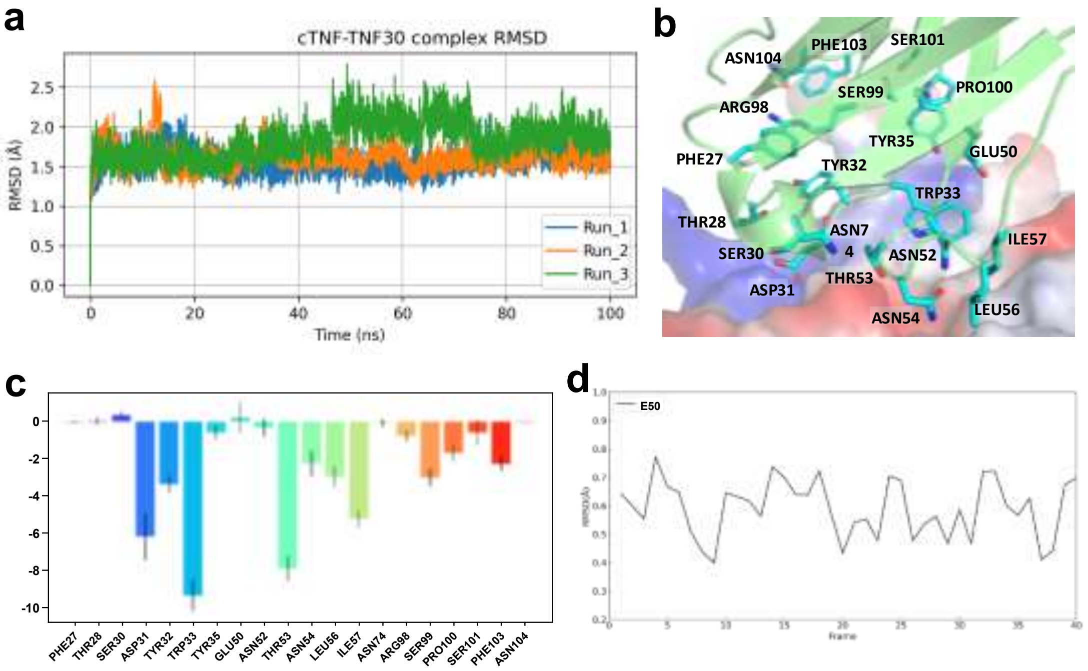

Supplementary Fig. 2: Molecule dynamics (MD) simulation based on cTNFα-TNF30 complex model and energetic differences of the residue contributions to the binding free energies. a The RMSD trajectory of cTNFα-TNF30 with three replicates. **b** TNF30 residues on the interface of cTNFα-TNF30 model. TNF30 is shown in cartoon model, and its residues on the interface of TNF30 and cTNFα are shown with side chains in cyan color, and cTNFα is shown with colored surface model. **c** Per-residue energy decomposition analysis on the binding free energy within TNF30 residues on the interface of cTNFα and TNF30. **d** The RMSD plots of Glu50 in TNF30 within the last 40 frames in 2nd run of MD simulation based on cTNFα-TNF30 complex model.

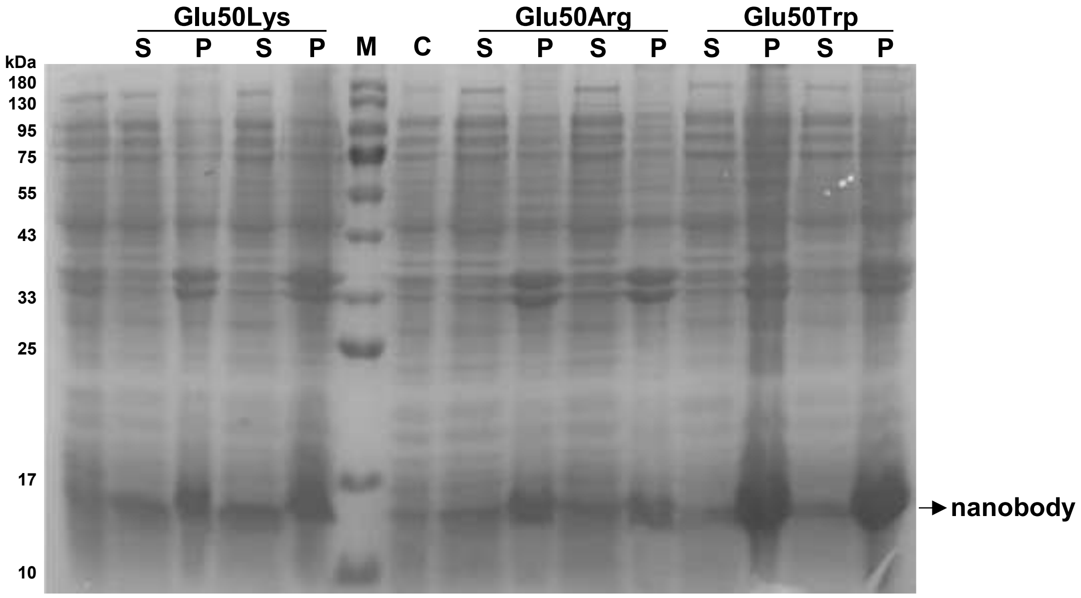

Supplementary Fig. 3: SDS-PAGE analysis of TNF30 mutants expressed in *E.coli*. For each mutant, 10 ml of *E. coli* culture was induced with IPTG and cultured in 16°C for 16 hrs. Harvested cells were lysed and centrifuged to get soluble fraction in supernatant (‘S’) and insoluble fraction in precipitation (‘P’). ‘M’ represents the protein marker, and the control samples with no IPTG added were labeled with ‘C’. Each mutation has two replicates for small scale expression test.

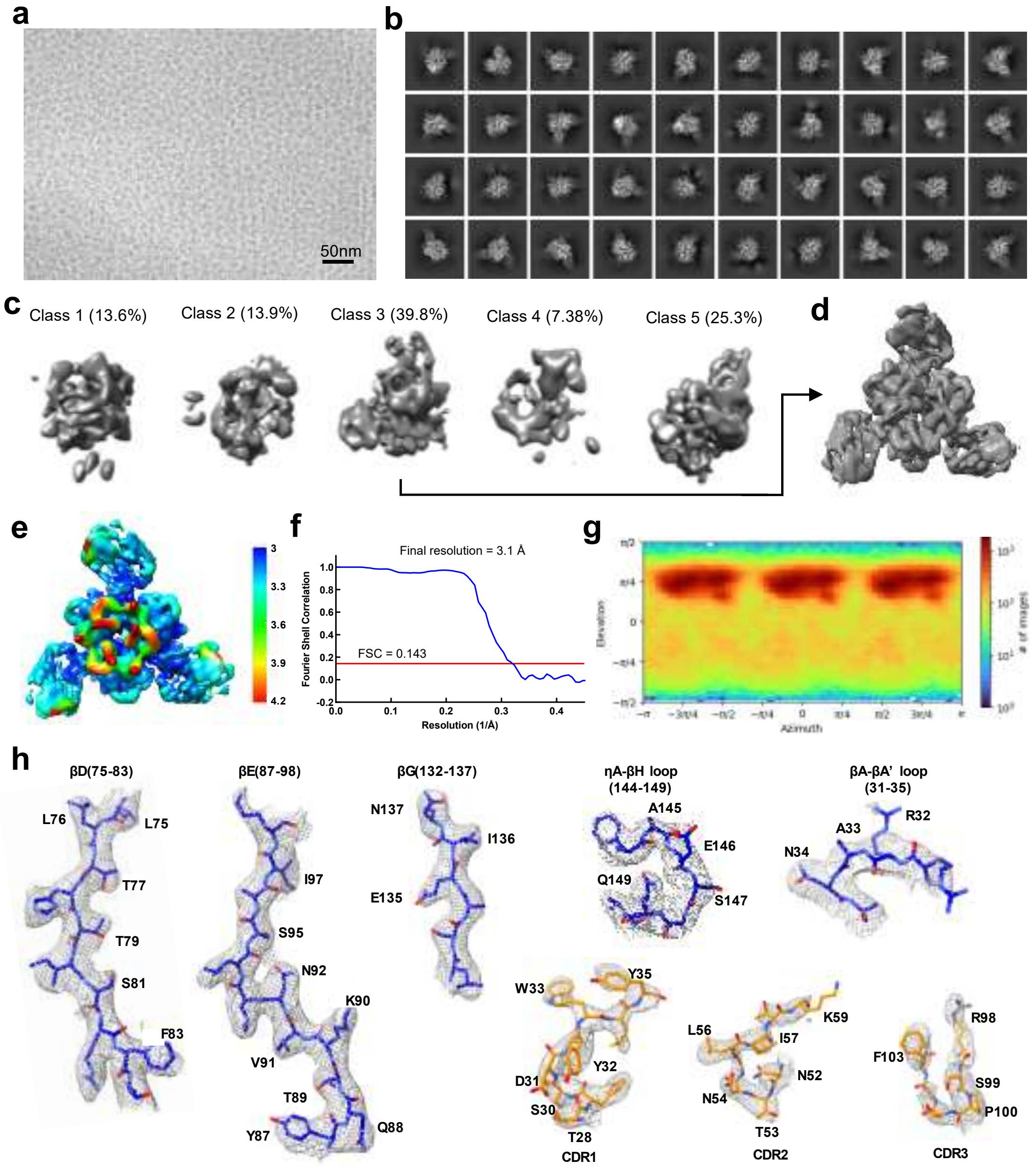

Supplementary Fig. 4: Cryo-EM reconstruction of the cTNFα with TNF30K. a A representative cryo-EM micrograph. **b** Representative 2D class averages. **c** 3D classes resolved in 3D classification. **d** Final map of cTNFα-TNF30K. **e** Local resolution analysis of the reconstructed map. **f** Orientation distribution of particles for the final map reconstruction. **g** The Fourier Shell Correlation (FSC) curve of the reconstruction. The resolution of the reconstruction was estimated using the gold-standard cutoff of FSC=0.143. **h** Cryo-EM density of cTNFα (blue) and TNF30K (orange) with atomic model fitted in. Labeled residues are those within the interface of cTNFα and TNF30K.

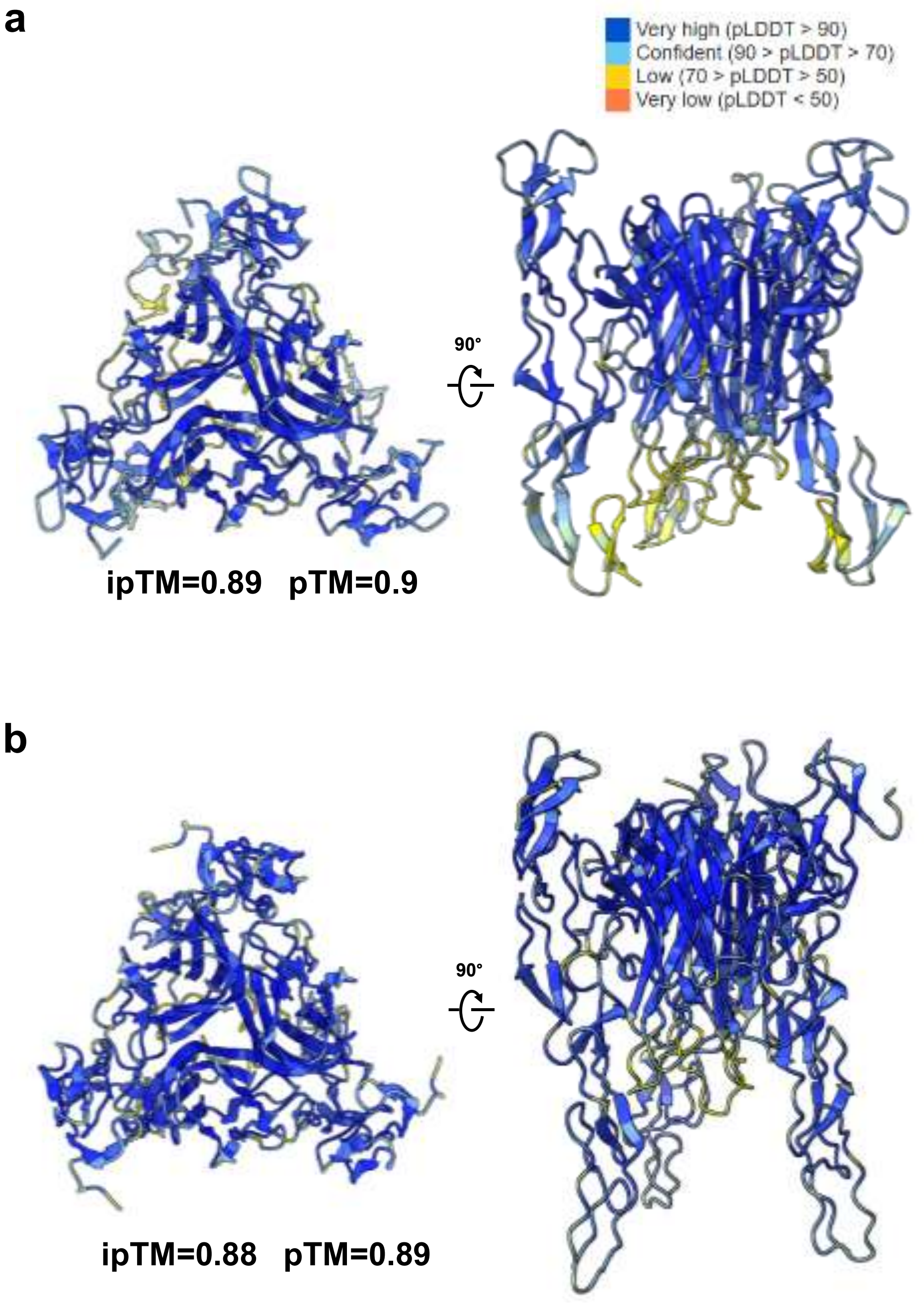

Supplementary Fig. 5: Predicted complex structures of cTNFα-cTNFR1 (a) and cTNFα-cTNFR2 (b) by Alphafold 3. Top and side views are shown in cartoon mode with colors indicating high confidence for most residues with pLDDT > 90, pTM > 0.5, ipTM > 0.8.

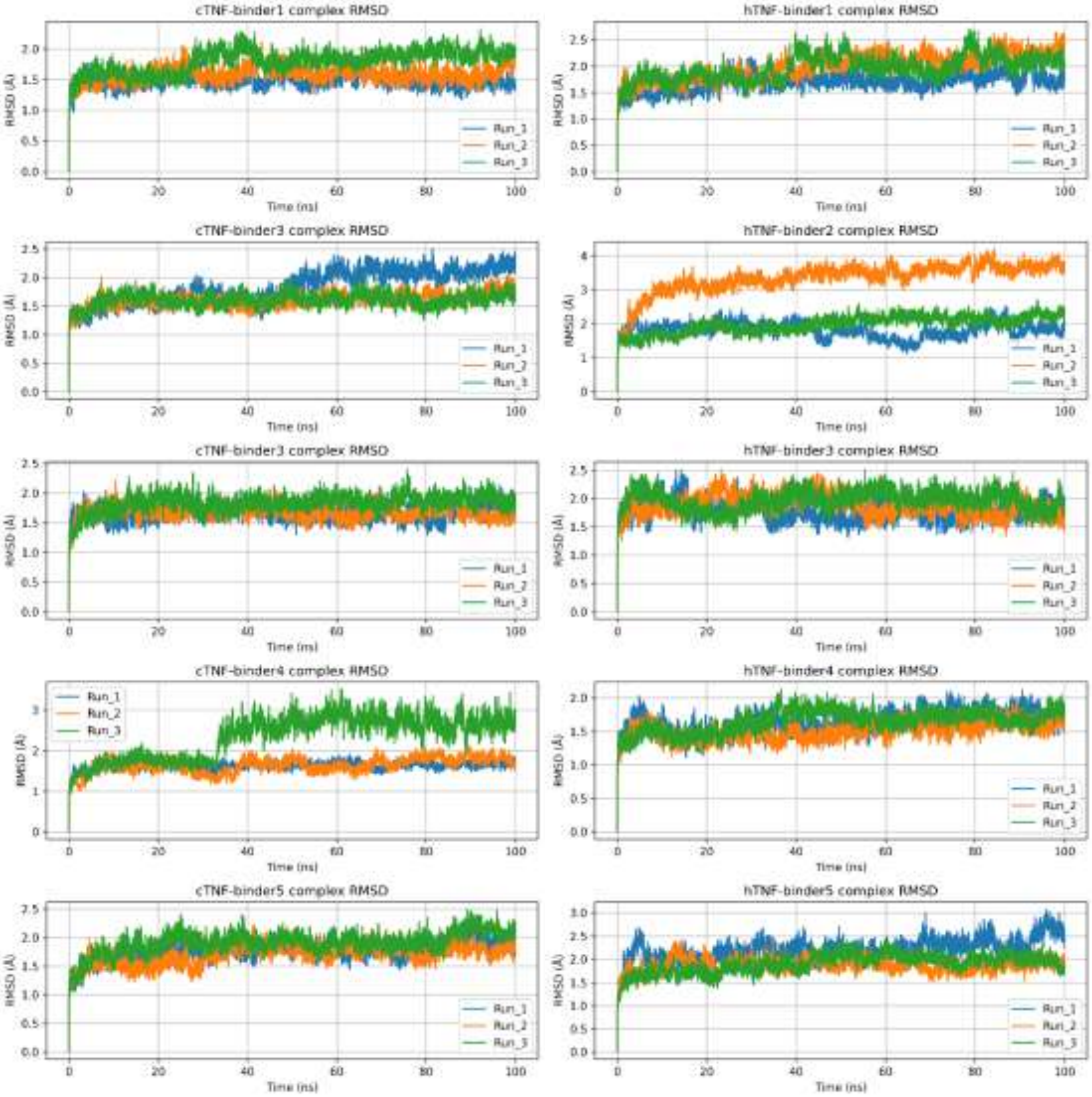

Supplementary Fig. 6: The RMSD trajectories of the cTNFα or hTNFα complexed with each designed minibinders. Each complex had three replicates.

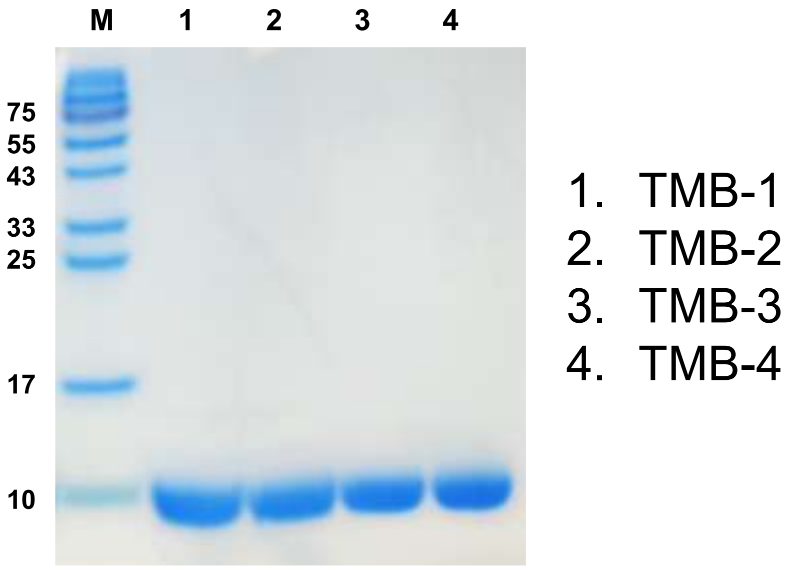

Supplementary Fig. 7: SDS-PAGE analysis of purified TNF minibinder proteins (TMB-1 to TMB-4). SEC purified minibinders TMB1 to TMB-4 were analyzed in a 15% SDS-PAGE gel.

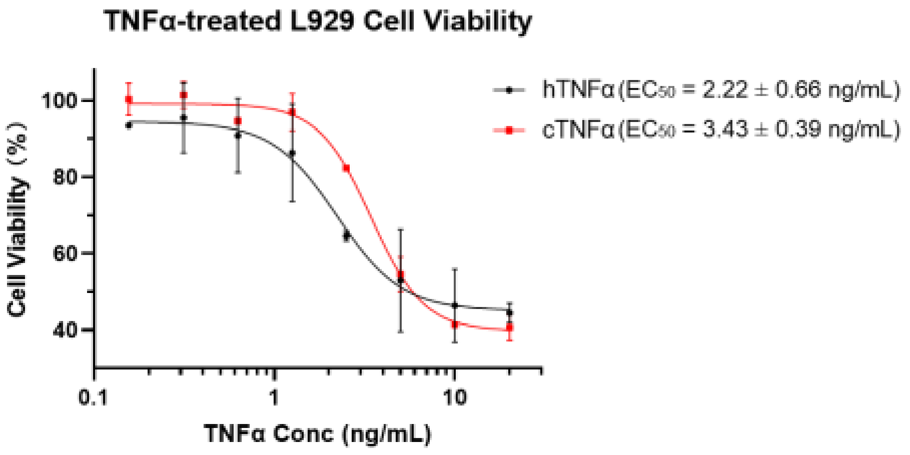

Supplementary Fig. 8: Human and canine TNFα-mediated cell death of L929 cell line. L929 cells in 96 well plate were treated with a serial dilution of hTNFα or cTNFα (0.156 to 200 ng/ml) and 1 μg/ml of actinomycin D. Cell viability was determined by CCK8 kit. Dose response curves and EC_{50} values were calculated with GraphPad Prism. EC_{50} values were reported as mean ± SD of three independent experiments (n=3).

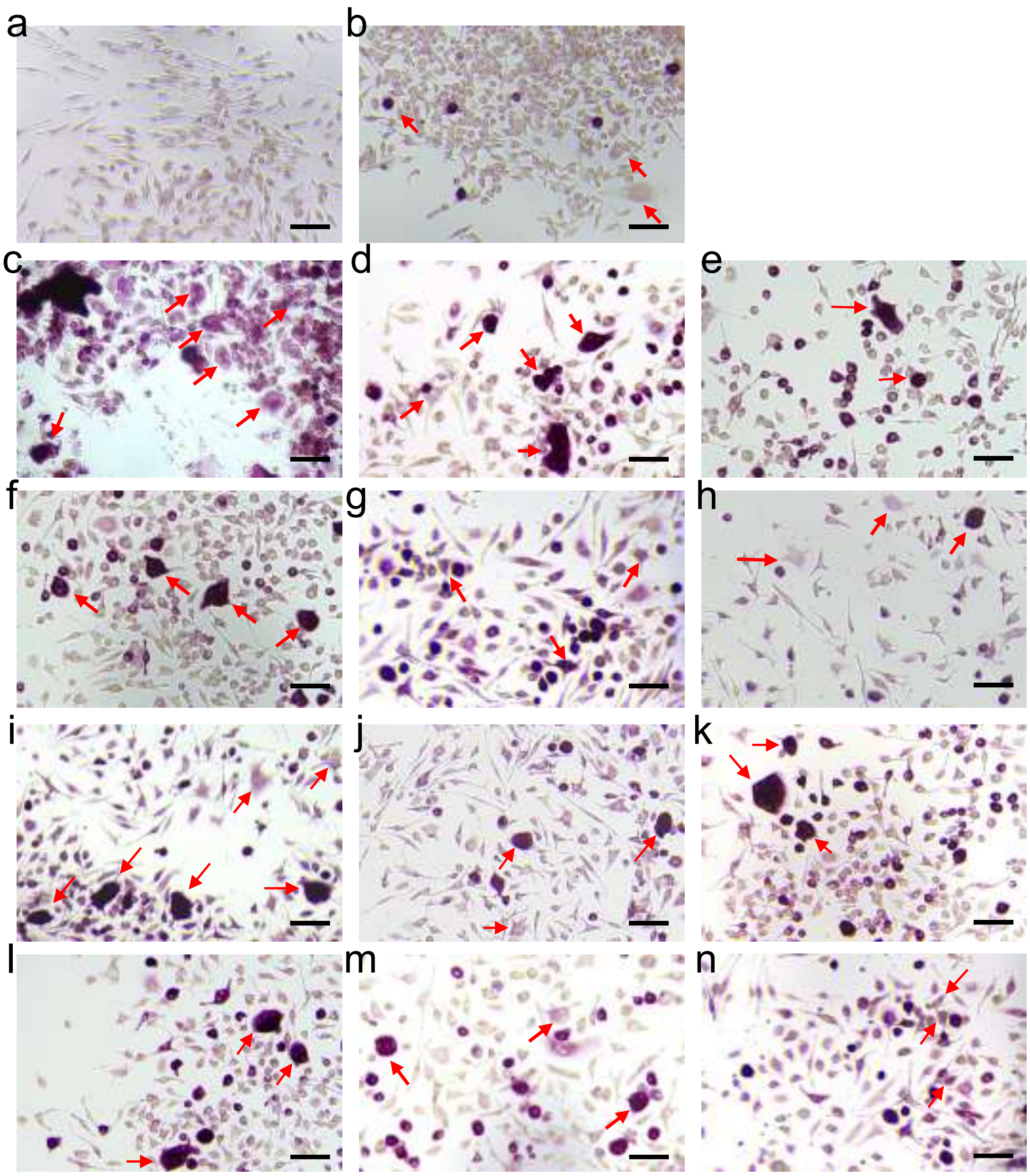

Supplementary Fig. 9: Representive TRAP staining images of primary bone marrow-derived macrophages (BMDMs) cells treated with TNFα and antagonists. a,b Primary BMDM cells treated with M-CSF (**a**), or with M-CSF and RANKL (**b**). **c-h** Primary BMDM cells treated with M-CSF, RANKL and cTNFα (**c**), plus TNF30 (**d**), TMB-1 (**e**), TMB-2 (**f**), TMB-3 (**g**) or TMB-4 (**h**). **i-n** Primary BMDM cells treated with M-CSF, RANKL and hTNFα (**i**), plus TNF30 (**j**), TMB-1 (**k**), TMB-2 (**l**), TMB-3 (**m**) or TMB-4 (**n**). Red arrows point the TRAP staining-positive multinucleated cells with at least 3 nuclei. Scale bar, 100 μm.

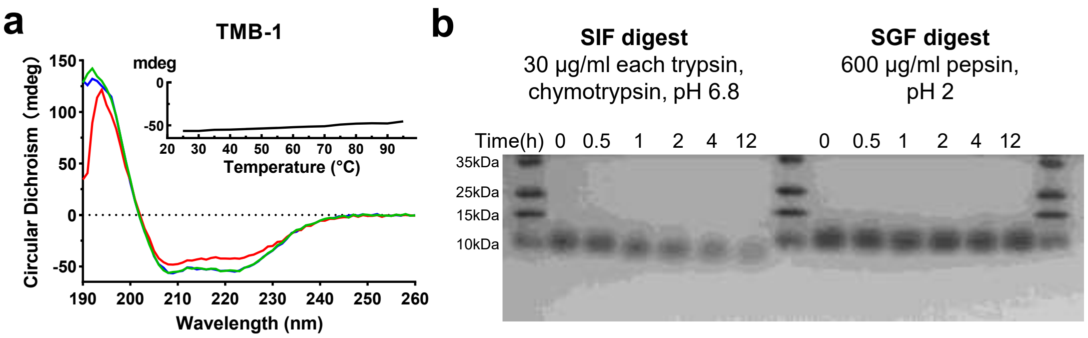

Supplementary Fig. 10: Thermostability and *in vitro* stability of TMB-1. a Circular dichroism spectra were recorded at varying temperatures (green, 25 °C; red, 95 °C; blue, 95 °C followed by cooling to 25 °C) on a JASCO-1700 circular dichroism spectrometer with a temperature-controlled cell holder. Protein samples were prepared at 0.1-0.2 mg/ml in potassium phosphate buffer. The dichroism signal at 222 nm of TMB-1 slightly increased upon raising temperature from 25 °C to 95 °C, and retained more than half of the mean residue ellipticity values at 95 °C, indicating that the melting temperature (Tm) value for TMB-1 is >95 °C. **b** Simulated intestinal and gastric fluids (SIF and SGF) digestion of TMB-1 at 37 °C for up to 12h (N ≥ 2). SIF was prepared as previously described, with trypsin and chymotrypsin each at 30 µg/mL[9]. SGF contained 34.2 mM NaCl and 0.6 mg/mL pepsin (add HCl to adjust pH to 2). Purified TMB-1 proteins were added to stock SGF and SIF solutions to a final concentration of 1 mg/mL. At each specified time point, aliquots were taken, mixed with load dye immediately and boiled at 95 °C for 10 minutes to quench protease activity. Digested samples were analyzed by SDS-PAGE using 4-20% Tris-glycine polyacrylamide gels.

Supplementary Table 1. Interface residues of canine TNFα in the complex with cTNFR1 and cTNFR2. PISA program (https://www.ebi.ac.uk/pdbe/pisa/) is applied based on the Alphafold 3 predicted complex structures. Residues with light green background represent those could interact with both receptors.

cTNFα	Residue	cTNFR1	cTNFR2	cTNFα	Residue	cTNFR1	cTNFR2
20	PRO	R1	R2	87	TYR	R1	R2
21	GLU	R1	R2	88	GLN	R1	R2
29	LEU	R1	R2	89	THR	R1	R2
30	SER	R1	R2	90	LYS		R2
31	ARG	R1	R2	91	VAL	R1	R2
32	ARG	R1	R2	92	ASN		R2
33	ALA	R1	R2	95	SER		R2
34	ASN	R1	R2	97	ILE	R1	R2
35	ALA	R1	R2	111	ALA	R1	
53	ASP	R1	R2	112	LYS	R1	R2
63	LEU	R1	R2	113	PRO	R1	R2
65	LYS	R1	R2	115	TYR	R1	R2
67	GLN	R1	R2	125	GLN	R1	
71	SER		R2	127	GLU	R1	R2
72	THR	R1	R2	128	LYS		R2
73	HIS	R1	R2	136	ILE	R1	
74	VAL	R1		137	ASN	R1	R2
75	LEU	R1	R2	138	LEU	R1	
76	LEU	R1	R2	143	ASP	R1	R2
77	THR	R1	R2	144	PHE	R1	R2
82	ARG	R1		145	ALA	R1	R2
84	ALA	R1		146	GLU	R1	R2
85	VAL	R1	R2	147	SER	R1	R2
86	SER	R1	R2	149	GLN	R1	R2

Supplementary Table 2. Interface residues of human TNFα in the complex with hTNFR1 and hTNFR2. PISA program (https://www.ebi.ac.uk/pdbe/pisa/) is applied based on the resolved complex structures of hTNFa-hTNFR1 (PDB 7KPB) and hTNFa-hTNFR2 (PDB 3ALQ). Residues with light green background represent those could interact with both receptors.

hTNFα	Residue	hTNFR1	hTNFR2	hTNFα	Residue	hTNFR1	hTNFR2
17	VAL	R1	R2	85	VAL	R1	R2
18	ALA	R1	R2	86	SER	R1	R2
20	PRO	R1	R2	87	TYR	R1	R2
21	GLN	R1	R2	88	GLN	R1	R2
23	GLU	R1	R2	89	THR	R1	R2
29	LEU	R1	R2	90	LYS	R1	R2
30	ASN	R1	R2	91	VAL	R1	R2
31	ARG	R1	R2	92	ASN	R1	R2
32	ARG	R1	R2	97	ILE	R1	R2
33	ALA	R1	R2	110	GLU	R1	
34	ASN	R1	R2	112	LYS	R1	
35	ALA	R1		113	PRO	R1	R2
53	GLU		R2	115	TYR	R1	R2
63	LEU	R1	R2	125	GLN		R2
65	LYS	R1		127	GLU	R1	R2
66	GLY	R1		128	PRO		R2
67	GLN	R1	R2	135	GLU	R1	R2
71	SER		R2	136	ILE	R1	
72	THR	R1	R2	137	ASN	R1	R2
73	HIS	R1	R2	138	ARG	R1	
74	VAL	R1		143	ASP	R1	R2
75	LEU	R1	R2	144	PHE	R1	R2
77	THR	R1	R2	145	ALA	R1	R2
81	SER		R2	146	GLU	R1	R2
82	ARG	R1	R2	147	SER	R1	R2
84	ALA	R1	R2	149	GLN	R1	R2

Supplementary Table 3 Top-5 computational designed minibinder proteins against cTNFα (TMB-1 to TMB-5)

Name	Sequence
TMB-1	SLDERLARELLEALRKITDPHEFETVVASIRILVKLARNPELERELREIEKRYREG
TMB-2	DTERQTVKILLHLARAAARLGDDRDVRTLLRLARELAKRNNDPELERLVREVEEEL
TMB-3	SEEEEERAIELFAEALNARLRGDPEEAKRLLEELERFAEETNDPAARTLVRLLREHL
TMB-4	DRELELLARIVVEALVETFNLSEEDKKYLEEVAKRLLEEGFSKDEIMQILSREAERLA
TMB-5	DEVRRLAEELEEDFKELLEKGDEELLRLVLQTLRILAQQYKDDEVKRILERLERLLR

Supplementary Table 4 Calculated binding energies of designed minibinders with cTNFα and hTNFα

	cTNFα		hTNFα	
	MM/GBSA (kcal/mol)	MM/PBSA (kcal/mol)	MM/GBSA (kcal/mol)	MM/PBSA (kcal/mol)
TMB-1	-45.06 ± 5.96*	-61.28 ± 6.66	-27.65 ± 7.00	-40.52 ± 8.66
TMB-2	-37.47 ± 7.49	52.44 ± 8.37	-9.88 ± 7.61	-19.31 ± 8.82
TMB-3	-30.19 ± 6.83	-40.79 ± 9.12	-33.27 ± 8.06	-29.46 ± 12.95
TMB-4	-43.94 ± 8.03	-46.63 ± 9.45	-5.08 ± 8.24	-7.43 ± 10.64
TMB-5	-43.92 ± 7.24	-60.32 ± 8.07	-12.05 ± 7.53	-9.04 ± 11.73

* All values were presented as mean ± SD.

Supplementary Table 5. Results of predicted immunogenicity of designed minibinders using NetMHCII software*.

HLA variant	TMB-1		TMB-2		TMB-3		TMB-4		TMB-5		TNF30	
	Strong	Weak	Strong	Weak	Strong	Weak	Strong	Weak	Strong	Weak	Strong	Weak
HLA-DPA10103-DPB10301	11	8	15	9	8	8	4	5	0	6	0	5
HLA-DPA10103-DPB10401	0	0	0	0	0	2	0	0	0	4	2	8
HLA-DPA10103-DPB10402	0	0	0	0	0	4	0	5	0	5	0	10
HLA-DPA10103-DPB10601	0	0	0	0	0	0	0	4	0	8	0	4
HLA-DPA10201-DPB10101	0	10	0	0	0	13	0	3	0	11	0	0
HLA-DPA10201-DPB10501	1	13	0	14	4	10	0	5	1	2	0	0
HLA-DPA10201-DPB11401	1	9	0	8	2	5	0	10	0	1	0	0
HLA-DPA10301-DPB10402	4	7	0	0	0	9	0	4	6	3	0	6
HLA-DPA10103-DPB10201	0	0	0	0	0	0	0	3	0	4	7	2
HLA-DQA10101-DQB10501	0	0	0	0	0	1	0	2	0	0	3	7
HLA-DQA10102-DQB10501	0	12	5	2	0	0	0	2	0	7	0	0
HLA-DQA10102-DQB10502	0	0	0	2	0	7	0	8	4	9	3	4
HLA-DQA10102-DQB10602	0	0	0	12	0	5	0	0	4	1	1	8
HLA-DQA10103-DQB10603	0	6	0	0	0	0	0	1	0	2	0	0
HLA-DQA10104-DQB10503	0	0	0	0	0	11	0	5	0	0	3	9
HLA-DQA10201-DQB10202	5	2	1	1	0	14	0	10	3	3	0	7
HLA-DQA10201-DQB10301	0	6	0	0	0	0	0	0	0	0	2	15
HLA-DQA10201-DQB10303	0	5	0	6	0	0	0	0	0	0	0	3
HLA-DQA10201-DQB10402	0	5	4	3	0	2	0	0	0	1	1	21
HLA-DQA10301-DQB10301	0	0	0	6	0	0	0	0	0	0	4	18
HLA-DQA10301-DQB10302	0	0	0	2	6	7	3	8	0	2	0	1
HLA-DQA10303-DQB10402	2	7	11	7	0	9	0	3	0	0	10	11
HLA-DQA10401-DQB10402	0	8	2	2	8	10	6	13	0	3	0	0
HLA-DQA10501-DQB10201	0	0	1	1	0	8	0	8	2	3	0	1
HLA-DQA10501-DQB10301	0	0	0	0	0	0	0	0	0	0	6	14
HLA-DQA10501-DQB10302	0	0	0	4	0	0	0	0	0	0	0	14
HLA-DQA10501-DQB10303	0	0	0	4	0	0	0	0	0	0	0	13
HLA-DQA10501-DQB10402	0	7	8	2	0	4	0	0	0	4	14	2
HLA-DQA10601-DQB10402	0	8	3	4	0	0	0	0	0	6	9	11
DRB1_0101	0	0	3	4	0	6	0	0	0	0	4	22
DRB1_0103	9	3	7	6	1	2	0	5	13	4	0	6
DRB1_0301	3	9	0	12	0	1	0	0	0	8	0	0
DRB1_0401	0	4	0	0	0	3	0	4	0	4	16	13
DRB1_0402	0	0	2	9	0	0	0	0	0	0	0	7
DRB1_0403	0	1	0	5	0	0	0	7	0	4	0	2
DRB1_0404	0	10	3	4	0	0	0	0	3	4	0	10
DRB1_0405	1	4	0	3	0	6	0	7	0	5	6	5
DRB1_0701	0	6	0	2	0	0	0	2	0	1	0	0
DRB1_0801	7	8	13	8	0	3	0	6	0	11	0	7
DRB1_0802	2	10	6	8	0	0	0	0	2	4	0	6
DRB1_0901	0	0	0	6	0	1	0	0	0	0	5	7
DRB1_1001	0	0	0	0	0	2	0	0	0	6	5	2
DRB1_1101	6	9	14	6	0	3	0	5	0	4	0	0
DRB1_1201	6	7	4	5	0	1	0	0	5	7	0	0
DRB1_1301	4	15	13	9	1	5	0	5	8	4	0	0
DRB1_1302	0	3	0	3	0	0	0	0	0	0	0	9
DRB1_1501	0	0	0	5	0	4	0	3	0	0	4	5
DRB1_1602	0	7	0	2	0	0	0	0	0	0	0	12
DRB3_0101	0	0	0	0	0	0	0	11	5	1	3	10
DRB3_0202	0	4	0	3	0	0	0	0	0	0	0	12
DRB3_0301	0	0	0	3	0	0	0	0	0	4	0	9
DRB4_0101	0	2	0	15	0	4	0	0	13	4	7	8
DRB4_0103	3	8	16	7	3	1	0	6	7	6	0	11
DRB5_0101	0	16	6	6	0	5	0	0	0	0	0	11

*The immunogenicity of designed minibinders and comparator TNF30 were evaluated by predicting the binding of peptides derived from designed minibinders and comparator TNF30 to the most common

MHCII variants, including 9 HLA-DP alleles, 20 HLA-DQ alleles, and 25 HLA-DR alleles. NetMHCII software (v2.3) was applied, and predicted results were ranked as strong (top 2%), weak (top 2-10%), and non-binding (lower 90%). The number of hits for strong (red) and weak (blue) binders are listed below and colored from no binding peptides (white) to maximum number of binding peptides (red/blue). Designed minibinders present lower predicted immunogenicity than TNF30, a humanized nanobody that did not elicit strong anti-drug response in mice and humans[10,11].

Supplementary Table 6 Detailed parameters for each MD simulation system

System	Repeat	Box Size (nm)	All Atoms	Water Molecules	Salinity
cTNF-TNF30	3 (100 ns)	8.588×9.291×9.678	76,907	22,760	0
cTNF-binder1	3 (100 ns)	7.797×8.178×8.152	52,285	14,868	0
cTNF-binder2	3 (100 ns)	7.808×8.291×8.164	52,935	15,094	0
cTNF-binder3	3 (100 ns)	7.813×8.234×8.169	52,580	14,953	0
cTNF-binder4	3 (100 ns)	7.800×8.102×8.155	51,680	14,678	0
cTNF-binder5	3 (100 ns)	7.797×8.272×8.153	52,681	15,002	0
hTNF-binder1	3 (100 ns)	7.526×7.747×7.940	46,202	12,896	0
hTNF-binder2	3 (100 ns)	8.170×7.510×7.723	47,508	13,342	0
hTNF-binder3	3 (100 ns)	8.373×7.503×7.645	48,136	13,528	0
hTNF-binder4	3 (100 ns)	8.343×7.497×7.631	47,813	13,446	0
hTNF-binder5	3 (100 ns)	8.704×7.502×7.658	50,130	14,208	0

Supplementary References:

1. Abraham MJ, *et al*. GROMACS: High performance molecular simulations through multi-level parallelism from laptops to supercomputers. SoftwareX 1, 19-25 (2015).
2. Jorgensen WL, *et al*. Comparison of Simple Potential Functions for Simulating Liquid Water, J Chem Phys 79, 926–935 (1983).
3. Price DJ, Brooks CL III. A modified **TIP3P** water potential for simulation with Ewald summation. J Chem Phys 121(20), 10096-103 (2004).
4. Bussi G, *et al*. Canonical sampling through velocity rescaling, J Chem Phys 126(1), 014101 (2007).
5. Parrinello M, Rahman A. Crystal structure and pair potentials: A molecular- dynamics study, Phys. Rev. Lett. 45, 1196–1199 (1980).
6. Hess B, *et al*. LINCS: A Linear Constraint Solver for Molecular Simulations. J Comput Chem 18, 1463–1472 (1997).
7. Essmann U, *et al*. Pedersen, A Smooth Particle Mesh Ewald Method. J Chem Phys 103, 8577–8593 (1995).
8. Cheatham TE III, *et al*. Molecular Dynamics Simulations on Solvated Biomolecular Systems: The Particle Mesh Ewald Method Leads to Stable Trajectories of DNA, RNA, and Protein. J Am Chem Soc 117, 4193–4194 (1995).
9. Berger S, *et al*. Preclinical proof of principle for orally delivered Th17 antagonist miniproteins. Cell 187, 4305-4317.e4318 (2024).
10. Ishiwatari-Ogata C, *et al*. Ozoralizumab, a Humanized Anti-TNFα NANOBODY® Compound, Exhibits Efficacy Not Only at the Onset of Arthritis in a Human TNF Transgenic Mouse but Also During Secondary Failure of Administration of an Anti-TNFα IgG. Front Immunol 13, 853008 (2022).
11. Takeuchi T, *et al*. Efficacy and pharmacokinetics of ozoralizumab, an anti-TNFα NANOBODY® compound, in patients with rheumatoid arthritis: 52-week results from the OHZORA and NATSUZORA trials. Arthritis Research & Therapy 25, 60 (2023).
